# A measurement of the ratio of the production cross sections for $W$ and $Z$ bosons in association with jets with the ATLAS detector

**DOI:** 10.1140/epjc/s10052-014-3168-9

**Published:** 2014-12-02

**Authors:** ATLAS Collaboration, G. Aad, B. Abbott, J. Abdallah, S. Abdel Khalek, O. Abdinov, R. Aben, B. Abi, M. Abolins, O. S. AbouZeid, H. Abramowicz, H. Abreu, R. Abreu, Y. Abulaiti, B. S. Acharya, L. Adamczyk, D. L. Adams, J. Adelman, S. Adomeit, T. Adye, T. Agatonovic-Jovin, J. A. Aguilar-Saavedra, M. Agustoni, S. P. Ahlen, F. Ahmadov, G. Aielli, H. Akerstedt, T. P. A. Åkesson, G. Akimoto, A. V. Akimov, G. L. Alberghi, J. Albert, S. Albrand, M. J. Alconada Verzini, M. Aleksa, I. N. Aleksandrov, C. Alexa, G. Alexander, G. Alexandre, T. Alexopoulos, M. Alhroob, G. Alimonti, L. Alio, J. Alison, B. M. M. Allbrooke, L. J. Allison, P. P. Allport, J. Almond, A. Aloisio, A. Alonso, F. Alonso, C. Alpigiani, A. Altheimer, B. Alvarez Gonzalez, M. G. Alviggi, K. Amako, Y. Amaral Coutinho, C. Amelung, D. Amidei, S. P. Amor Dos Santos, A. Amorim, S. Amoroso, N. Amram, G. Amundsen, C. Anastopoulos, L. S. Ancu, N. Andari, T. Andeen, C. F. Anders, G. Anders, K. J. Anderson, A. Andreazza, V. Andrei, X. S. Anduaga, S. Angelidakis, I. Angelozzi, P. Anger, A. Angerami, F. Anghinolfi, A. V. Anisenkov, N. Anjos, A. Annovi, A. Antonaki, M. Antonelli, A. Antonov, J. Antos, F. Anulli, M. Aoki, L. Aperio Bella, R. Apolle, G. Arabidze, I. Aracena, Y. Arai, J. P. Araque, A. T. H. Arce, J.-F. Arguin, S. Argyropoulos, M. Arik, A. J. Armbruster, O. Arnaez, V. Arnal, H. Arnold, M. Arratia, O. Arslan, A. Artamonov, G. Artoni, S. Asai, N. Asbah, A. Ashkenazi, B. Åsman, L. Asquith, K. Assamagan, R. Astalos, M. Atkinson, N. B. Atlay, B. Auerbach, K. Augsten, M. Aurousseau, G. Avolio, G. Azuelos, Y. Azuma, M. A. Baak, A. E. Baas, C. Bacci, H. Bachacou, K. Bachas, M. Backes, M. Backhaus, J. Backus Mayes, E. Badescu, P. Bagiacchi, P. Bagnaia, Y. Bai, T. Bain, J. T. Baines, O. K. Baker, P. Balek, F. Balli, E. Banas, Sw. Banerjee, A. A. E. Bannoura, V. Bansal, H. S. Bansil, L. Barak, S. P. Baranov, E. L. Barberio, D. Barberis, M. Barbero, T. Barillari, M. Barisonzi, T. Barklow, N. Barlow, B. M. Barnett, R. M. Barnett, Z. Barnovska, A. Baroncelli, G. Barone, A. J. Barr, F. Barreiro, J. Barreiro Guimarães da Costa, R. Bartoldus, A. E. Barton, P. Bartos, V. Bartsch, A. Bassalat, A. Basye, R. L. Bates, J. R. Batley, M. Battaglia, M. Battistin, F. Bauer, H. S. Bawa, M. D. Beattie, T. Beau, P. H. Beauchemin, R. Beccherle, P. Bechtle, H. P. Beck, K. Becker, S. Becker, M. Beckingham, C. Becot, A. J. Beddall, A. Beddall, S. Bedikian, V. A. Bednyakov, C. P. Bee, L. J. Beemster, T. A. Beermann, M. Begel, K. Behr, C. Belanger-Champagne, P. J. Bell, W. H. Bell, G. Bella, L. Bellagamba, A. Bellerive, M. Bellomo, K. Belotskiy, O. Beltramello, O. Benary, D. Benchekroun, K. Bendtz, N. Benekos, Y. Benhammou, E. Benhar Noccioli, J. A. Benitez Garcia, D. P. Benjamin, J. R. Bensinger, K. Benslama, S. Bentvelsen, D. Berge, E. Bergeaas Kuutmann, N. Berger, F. Berghaus, J. Beringer, C. Bernard, P. Bernat, C. Bernius, F. U. Bernlochner, T. Berry, P. Berta, C. Bertella, G. Bertoli, F. Bertolucci, C. Bertsche, D. Bertsche, M. F. Bessner, M. I. Besana, G. J. Besjes, O. Bessidskaia, N. Besson, C. Betancourt, S. Bethke, W. Bhimji, R. M. Bianchi, L. Bianchini, M. Bianco, O. Biebel, S. P. Bieniek, K. Bierwagen, J. Biesiada, M. Biglietti, J. Bilbao De Mendizabal, H. Bilokon, M. Bindi, S. Binet, A. Bingul, C. Bini, C. W. Black, J. E. Black, K. M. Black, D. Blackburn, R. E. Blair, J.-B. Blanchard, T. Blazek, I. Bloch, C. Blocker, W. Blum, U. Blumenschein, G. J. Bobbink, V. S. Bobrovnikov, S. S. Bocchetta, A. Bocci, C. Bock, C. R. Boddy, M. Boehler, J. Boek, J. Boek, T. T. Boek, J. A. Bogaerts, A. G. Bogdanchikov, A. Bogouch, C. Bohm, J. Bohm, V. Boisvert, T. Bold, V. Boldea, A. S. Boldyrev, M. Bomben, M. Bona, M. Boonekamp, A. Borisov, G. Borissov, M. Borri, S. Borroni, J. Bortfeldt, V. Bortolotto, K. Bos, D. Boscherini, M. Bosman, H. Boterenbrood, J. Boudreau, J. Bouffard, E. V. Bouhova-Thacker, D. Boumediene, C. Bourdarios, N. Bousson, S. Boutouil, A. Boveia, J. Boyd, I. R. Boyko, I. Bozic, J. Bracinik, A. Brandt, G. Brandt, O. Brandt, U. Bratzler, B. Brau, J. E. Brau, H. M. Braun, S. F. Brazzale, B. Brelier, K. Brendlinger, A. J. Brennan, R. Brenner, S. Bressler, K. Bristow, T. M. Bristow, D. Britton, F. M. Brochu, I. Brock, R. Brock, C. Bromberg, J. Bronner, G. Brooijmans, T. Brooks, W. K. Brooks, J. Brosamer, E. Brost, J. Brown, P. A. Bruckman de Renstrom, D. Bruncko, R. Bruneliere, S. Brunet, A. Bruni, G. Bruni, M. Bruschi, L. Bryngemark, T. Buanes, Q. Buat, F. Bucci, P. Buchholz, R. M. Buckingham, A. G. Buckley, S. I. Buda, I. A. Budagov, F. Buehrer, L. Bugge, M. K. Bugge, O. Bulekov, A. C. Bundock, H. Burckhart, S. Burdin, B. Burghgrave, S. Burke, I. Burmeister, E. Busato, D. Büscher, V. Büscher, P. Bussey, C. P. Buszello, B. Butler, J. M. Butler, A. I. Butt, C. M. Buttar, J. M. Butterworth, P. Butti, W. Buttinger, A. Buzatu, M. Byszewski, S. Cabrera Urbán, D. Caforio, O. Cakir, P. Calafiura, A. Calandri, G. Calderini, P. Calfayan, R. Calkins, L. P. Caloba, D. Calvet, S. Calvet, R. Camacho Toro, S. Camarda, D. Cameron, L. M. Caminada, R. Caminal Armadans, S. Campana, M. Campanelli, A. Campoverde, V. Canale, A. Canepa, M. Cano Bret, J. Cantero, R. Cantrill, T. Cao, M. D. M. Capeans Garrido, I. Caprini, M. Caprini, M. Capua, R. Caputo, R. Cardarelli, T. Carli, G. Carlino, L. Carminati, S. Caron, E. Carquin, G. D. Carrillo-Montoya, J. R. Carter, J. Carvalho, D. Casadei, M. P. Casado, M. Casolino, E. Castaneda-Miranda, A. Castelli, V. Castillo Gimenez, N. F. Castro, P. Catastini, A. Catinaccio, J. R. Catmore, A. Cattai, G. Cattani, J. Caudron, V. Cavaliere, D. Cavalli, M. Cavalli-Sforza, V. Cavasinni, F. Ceradini, B. C. Cerio, K. Cerny, A. S. Cerqueira, A. Cerri, L. Cerrito, F. Cerutti, M. Cerv, A. Cervelli, S. A. Cetin, A. Chafaq, D. Chakraborty, I. Chalupkova, P. Chang, B. Chapleau, J. D. Chapman, D. Charfeddine, D. G. Charlton, C. C. Chau, C. A. Chavez Barajas, S. Cheatham, A. Chegwidden, S. Chekanov, S. V. Chekulaev, G. A. Chelkov, M. A. Chelstowska, C. Chen, H. Chen, K. Chen, L. Chen, S. Chen, X. Chen, Y. Chen, Y. Chen, H. C. Cheng, Y. Cheng, A. Cheplakov, R. Cherkaoui El Moursli, V. Chernyatin, E. Cheu, L. Chevalier, V. Chiarella, G. Chiefari, J. T. Childers, A. Chilingarov, G. Chiodini, A. S. Chisholm, R. T. Chislett, A. Chitan, M. V. Chizhov, S. Chouridou, B. K. B. Chow, D. Chromek-Burckhart, M. L. Chu, J. Chudoba, J. J. Chwastowski, L. Chytka, G. Ciapetti, A. K. Ciftci, R. Ciftci, D. Cinca, V. Cindro, A. Ciocio, P. Cirkovic, Z. H. Citron, M. Citterio, M. Ciubancan, A. Clark, P. J. Clark, R. N. Clarke, W. Cleland, J. C. Clemens, C. Clement, Y. Coadou, M. Cobal, A. Coccaro, J. Cochran, L. Coffey, J. G. Cogan, J. Coggeshall, B. Cole, S. Cole, A. P. Colijn, J. Collot, T. Colombo, G. Colon, G. Compostella, P. Conde Muiño, E. Coniavitis, M. C. Conidi, S. H. Connell, I. A. Connelly, S. M. Consonni, V. Consorti, S. Constantinescu, C. Conta, G. Conti, F. Conventi, M. Cooke, B. D. Cooper, A. M. Cooper-Sarkar, N. J. Cooper-Smith, K. Copic, T. Cornelissen, M. Corradi, F. Corriveau, A. Corso-Radu, A. Cortes-Gonzalez, G. Cortiana, G. Costa, M. J. Costa, D. Costanzo, D. Côté, G. Cottin, G. Cowan, B. E. Cox, K. Cranmer, G. Cree, S. Crépé-Renaudin, F. Crescioli, W. A. Cribbs, M. Crispin Ortuzar, M. Cristinziani, V. Croft, G. Crosetti, C.-M. Cuciuc, T. Cuhadar Donszelmann, J. Cummings, M. Curatolo, C. Cuthbert, H. Czirr, P. Czodrowski, Z. Czyczula, S. D’Auria, M. D’Onofrio, M. J. Da Cunha Sargedas De Sousa, C. Da Via, W. Dabrowski, A. Dafinca, T. Dai, O. Dale, F. Dallaire, C. Dallapiccola, M. Dam, A. C. Daniells, M. Dano Hoffmann, V. Dao, G. Darbo, S. Darmora, J. A. Dassoulas, A. Dattagupta, W. Davey, C. David, T. Davidek, E. Davies, M. Davies, O. Davignon, A. R. Davison, P. Davison, Y. Davygora, E. Dawe, I. Dawson, R. K. Daya-Ishmukhametova, K. De, R. de Asmundis, S. De Castro, S. De Cecco, N. De Groot, P. de Jong, H. De la Torre, F. De Lorenzi, L. De Nooij, D. De Pedis, A. De Salvo, U. De Sanctis, A. De Santo, J. B. De Vivie De Regie, W. J. Dearnaley, R. Debbe, C. Debenedetti, B. Dechenaux, D. V. Dedovich, I. Deigaard, J. Del Peso, T. Del Prete, F. Deliot, C. M. Delitzsch, M. Deliyergiyev, A. Dell’Acqua, L. Dell’Asta, M. Dell’Orso, M. Della Pietra, D. della Volpe, M. Delmastro, P. A. Delsart, C. Deluca, S. Demers, M. Demichev, A. Demilly, S. P. Denisov, D. Derendarz, J. E. Derkaoui, F. Derue, P. Dervan, K. Desch, C. Deterre, P. O. Deviveiros, A. Dewhurst, S. Dhaliwal, A. Di Ciaccio, L. Di Ciaccio, A. Di Domenico, C. Di Donato, A. Di Girolamo, B. Di Girolamo, A. Di Mattia, B. Di Micco, R. Di Nardo, A. Di Simone, R. Di Sipio, D. Di Valentino, F. A. Dias, M. A. Diaz, E. B. Diehl, J. Dietrich, T. A. Dietzsch, S. Diglio, A. Dimitrievska, J. Dingfelder, C. Dionisi, P. Dita, S. Dita, F. Dittus, F. Djama, T. Djobava, M. A. B. do Vale, A. Do Valle Wemans, D. Dobos, C. Doglioni, T. Doherty, T. Dohmae, J. Dolejsi, Z. Dolezal, B. A. Dolgoshein, M. Donadelli, S. Donati, P. Dondero, J. Donini, J. Dopke, A. Doria, M. T. Dova, A. T. Doyle, M. Dris, J. Dubbert, S. Dube, E. Dubreuil, E. Duchovni, G. Duckeck, O. A. Ducu, D. Duda, A. Dudarev, F. Dudziak, L. Duflot, L. Duguid, M. Dührssen, M. Dunford, H. Duran Yildiz, M. Düren, A. Durglishvili, M. Dwuznik, M. Dyndal, J. Ebke, W. Edson, N. C. Edwards, W. Ehrenfeld, T. Eifert, G. Eigen, K. Einsweiler, T. Ekelof, M. El Kacimi, M. Ellert, S. Elles, F. Ellinghaus, N. Ellis, J. Elmsheuser, M. Elsing, D. Emeliyanov, Y. Enari, O. C. Endner, M. Endo, R. Engelmann, J. Erdmann, A. Ereditato, D. Eriksson, G. Ernis, J. Ernst, M. Ernst, J. Ernwein, D. Errede, S. Errede, E. Ertel, M. Escalier, H. Esch, C. Escobar, B. Esposito, A. I. Etienvre, E. Etzion, H. Evans, A. Ezhilov, L. Fabbri, G. Facini, R. M. Fakhrutdinov, S. Falciano, R. J. Falla, J. Faltova, Y. Fang, M. Fanti, A. Farbin, A. Farilla, T. Farooque, S. Farrell, S. M. Farrington, P. Farthouat, F. Fassi, P. Fassnacht, D. Fassouliotis, A. Favareto, L. Fayard, P. Federic, O. L. Fedin, W. Fedorko, M. Fehling-Kaschek, S. Feigl, L. Feligioni, C. Feng, E. J. Feng, H. Feng, A. B. Fenyuk, S. Fernandez Perez, S. Ferrag, J. Ferrando, A. Ferrari, P. Ferrari, R. Ferrari, D. E. Ferreira de Lima, A. Ferrer, D. Ferrere, C. Ferretti, A. Ferretto Parodi, M. Fiascaris, F. Fiedler, A. Filipčič, M. Filipuzzi, F. Filthaut, M. Fincke-Keeler, K. D. Finelli, M. C. N. Fiolhais, L. Fiorini, A. Firan, A. Fischer, J. Fischer, W. C. Fisher, E. A. Fitzgerald, M. Flechl, I. Fleck, P. Fleischmann, S. Fleischmann, G. T. Fletcher, G. Fletcher, T. Flick, A. Floderus, L. R. Flores Castillo, A. C. Florez Bustos, M. J. Flowerdew, A. Formica, A. Forti, D. Fortin, D. Fournier, H. Fox, S. Fracchia, P. Francavilla, M. Franchini, S. Franchino, D. Francis, L. Franconi, M. Franklin, S. Franz, M. Fraternali, S. T. French, C. Friedrich, F. Friedrich, D. Froidevaux, J. A. Frost, C. Fukunaga, E. Fullana Torregrosa, B. G. Fulsom, J. Fuster, C. Gabaldon, O. Gabizon, A. Gabrielli, A. Gabrielli, S. Gadatsch, S. Gadomski, G. Gagliardi, P. Gagnon, C. Galea, B. Galhardo, E. J. Gallas, V. Gallo, B. J. Gallop, P. Gallus, G. Galster, K. K. Gan, J. Gao, Y. S. Gao, F. M. Garay Walls, F. Garberson, C. García, J. E. García Navarro, M. Garcia-Sciveres, R. W. Gardner, N. Garelli, V. Garonne, C. Gatti, G. Gaudio, B. Gaur, L. Gauthier, P. Gauzzi, I. L. Gavrilenko, C. Gay, G. Gaycken, E. N. Gazis, P. Ge, Z. Gecse, C. N. P. Gee, D. A. A. Geerts, Ch. Geich-Gimbel, K. Gellerstedt, C. Gemme, A. Gemmell, M. H. Genest, S. Gentile, M. George, S. George, D. Gerbaudo, A. Gershon, H. Ghazlane, N. Ghodbane, B. Giacobbe, S. Giagu, V. Giangiobbe, P. Giannetti, F. Gianotti, B. Gibbard, S. M. Gibson, M. Gilchriese, T. P. S. Gillam, D. Gillberg, G. Gilles, D. M. Gingrich, N. Giokaris, M. P. Giordani, R. Giordano, F. M. Giorgi, F. M. Giorgi, P. F. Giraud, D. Giugni, C. Giuliani, M. Giulini, B. K. Gjelsten, S. Gkaitatzis, I. Gkialas, L. K. Gladilin, C. Glasman, J. Glatzer, P. C. F. Glaysher, A. Glazov, G. L. Glonti, M. Goblirsch-Kolb, J. R. Goddard, J. Godlewski, C. Goeringer, S. Goldfarb, T. Golling, D. Golubkov, A. Gomes, L. S. Gomez Fajardo, R. Gonçalo, J. Goncalves Pinto Firmino Da Costa, L. Gonella, S. González de la Hoz, G. Gonzalez Parra, S. Gonzalez-Sevilla, L. Goossens, P. A. Gorbounov, H. A. Gordon, I. Gorelov, B. Gorini, E. Gorini, A. Gorišek, E. Gornicki, A. T. Goshaw, C. Gössling, M. I. Gostkin, M. Gouighri, D. Goujdami, M. P. Goulette, A. G. Goussiou, C. Goy, S. Gozpinar, H. M. X. Grabas, L. Graber, I. Grabowska-Bold, P. Grafström, K.-J. Grahn, J. Gramling, E. Gramstad, S. Grancagnolo, V. Grassi, V. Gratchev, H. M. Gray, E. Graziani, O. G. Grebenyuk, Z. D. Greenwood, K. Gregersen, I. M. Gregor, P. Grenier, J. Griffiths, A. A. Grillo, K. Grimm, S. Grinstein, Ph. Gris, Y. V. Grishkevich, J.-F. Grivaz, J. P. Grohs, A. Grohsjean, E. Gross, J. Grosse-Knetter, G. C. Grossi, J. Groth-Jensen, Z. J. Grout, L. Guan, F. Guescini, D. Guest, O. Gueta, C. Guicheney, E. Guido, T. Guillemin, S. Guindon, U. Gul, C. Gumpert, J. Gunther, J. Guo, S. Gupta, P. Gutierrez, N. G. Gutierrez Ortiz, C. Gutschow, N. Guttman, C. Guyot, C. Gwenlan, C. B. Gwilliam, A. Haas, C. Haber, H. K. Hadavand, N. Haddad, P. Haefner, S. Hageböeck, Z. Hajduk, H. Hakobyan, M. Haleem, D. Hall, G. Halladjian, K. Hamacher, P. Hamal, K. Hamano, M. Hamer, A. Hamilton, S. Hamilton, G. N. Hamity, P. G. Hamnett, L. Han, K. Hanagaki, K. Hanawa, M. Hance, P. Hanke, R. Hann, J. B. Hansen, J. D. Hansen, P. H. Hansen, K. Hara, A. S. Hard, T. Harenberg, F. Hariri, S. Harkusha, D. Harper, R. D. Harrington, O. M. Harris, P. F. Harrison, F. Hartjes, M. Hasegawa, S. Hasegawa, Y. Hasegawa, A. Hasib, S. Hassani, S. Haug, M. Hauschild, R. Hauser, M. Havranek, C. M. Hawkes, R. J. Hawkings, A. D. Hawkins, T. Hayashi, D. Hayden, C. P. Hays, H. S. Hayward, S. J. Haywood, S. J. Head, T. Heck, V. Hedberg, L. Heelan, S. Heim, T. Heim, B. Heinemann, L. Heinrich, J. Hejbal, L. Helary, C. Heller, M. Heller, S. Hellman, D. Hellmich, C. Helsens, J. Henderson, Y. Heng, R. C. W. Henderson, C. Hengler, A. Henrichs, A. M. Henriques Correia, S. Henrot-Versille, C. Hensel, G. H. Herbert, Y. Hernández Jiménez, R. Herrberg-Schubert, G. Herten, R. Hertenberger, L. Hervas, G. G. Hesketh, N. P. Hessey, R. Hickling, E. Higón-Rodriguez, E. Hill, J. C. Hill, K. H. Hiller, S. Hillert, S. J. Hillier, I. Hinchliffe, E. Hines, M. Hirose, D. Hirschbuehl, J. Hobbs, N. Hod, M. C. Hodgkinson, P. Hodgson, A. Hoecker, M. R. Hoeferkamp, F. Hoenig, J. Hoffman, D. Hoffmann, J. I. Hofmann, M. Hohlfeld, T. R. Holmes, T. M. Hong, L. Hooft van Huysduynen, W. H. Hopkins, Y. Horii, J.-Y. Hostachy, S. Hou, A. Hoummada, J. Howard, J. Howarth, M. Hrabovsky, I. Hristova, J. Hrivnac, T. Hryn’ova, C. Hsu, P. J. Hsu, S.-C. Hsu, D. Hu, X. Hu, Y. Huang, Z. Hubacek, F. Hubaut, F. Huegging, T. B. Huffman, E. W. Hughes, G. Hughes, M. Huhtinen, T. A. Hülsing, M. Hurwitz, N. Huseynov, J. Huston, J. Huth, G. Iacobucci, G. Iakovidis, I. Ibragimov, L. Iconomidou-Fayard, E. Ideal, P. Iengo, O. Igonkina, T. Iizawa, Y. Ikegami, K. Ikematsu, M. Ikeno, Y. Ilchenko, D. Iliadis, N. Ilic, Y. Inamaru, T. Ince, P. Ioannou, M. Iodice, K. Iordanidou, V. Ippolito, A. Irles Quiles, C. Isaksson, M. Ishino, M. Ishitsuka, R. Ishmukhametov, C. Issever, S. Istin, J. M. Iturbe Ponce, R. Iuppa, J. Ivarsson, W. Iwanski, H. Iwasaki, J. M. Izen, V. Izzo, B. Jackson, M. Jackson, P. Jackson, M. R. Jaekel, V. Jain, K. Jakobs, S. Jakobsen, T. Jakoubek, J. Jakubek, D. O. Jamin, D. K. Jana, E. Jansen, H. Jansen, J. Janssen, M. Janus, G. Jarlskog, N. Javadov, T. Javůrek, L. Jeanty, J. Jejelava, G.-Y. Jeng, D. Jennens, P. Jenni, J. Jentzsch, C. Jeske, S. Jézéquel, H. Ji, J. Jia, Y. Jiang, M. Jimenez Belenguer, S. Jin, A. Jinaru, O. Jinnouchi, M. D. Joergensen, K. E. Johansson, P. Johansson, K. A. Johns, K. Jon-And, G. Jones, R. W. L. Jones, T. J. Jones, J. Jongmanns, P. M. Jorge, K. D. Joshi, J. Jovicevic, X. Ju, C. A. Jung, R. M. Jungst, P. Jussel, A. Juste Rozas, M. Kaci, A. Kaczmarska, M. Kado, H. Kagan, M. Kagan, E. Kajomovitz, C. W. Kalderon, S. Kama, A. Kamenshchikov, N. Kanaya, M. Kaneda, S. Kaneti, V. A. Kantserov, J. Kanzaki, B. Kaplan, A. Kapliy, D. Kar, K. Karakostas, N. Karastathis, M. J. Kareem, M. Karnevskiy, S. N. Karpov, Z. M. Karpova, K. Karthik, V. Kartvelishvili, A. N. Karyukhin, L. Kashif, G. Kasieczka, R. D. Kass, A. Kastanas, Y. Kataoka, A. Katre, J. Katzy, V. Kaushik, K. Kawagoe, T. Kawamoto, G. Kawamura, S. Kazama, V. F. Kazanin, M. Y. Kazarinov, R. Keeler, R. Kehoe, M. Keil, J. S. Keller, J. J. Kempster, H. Keoshkerian, O. Kepka, B. P. Kerševan, S. Kersten, K. Kessoku, J. Keung, F. Khalil-zada, H. Khandanyan, A. Khanov, A. Khodinov, A. Khomich, T. J. Khoo, G. Khoriauli, A. Khoroshilov, V. Khovanskiy, E. Khramov, J. Khubua, H. Y. Kim, H. Kim, S. H. Kim, N. Kimura, O. Kind, B. T. King, M. King, R. S. B. King, S. B. King, J. Kirk, A. E. Kiryunin, T. Kishimoto, D. Kisielewska, F. Kiss, T. Kittelmann, K. Kiuchi, E. Kladiva, M. Klein, U. Klein, K. Kleinknecht, P. Klimek, A. Klimentov, R. Klingenberg, J. A. Klinger, T. Klioutchnikova, P. F. Klok, E.-E. Kluge, P. Kluit, S. Kluth, E. Kneringer, E. B. F. G. Knoops, A. Knue, D. Kobayashi, T. Kobayashi, M. Kobel, M. Kocian, P. Kodys, P. Koevesarki, T. Koffas, E. Koffeman, L. A. Kogan, S. Kohlmann, Z. Kohout, T. Kohriki, T. Koi, H. Kolanoski, I. Koletsou, J. Koll, A. A. Komar, Y. Komori, T. Kondo, N. Kondrashova, K. Köneke, A. C. König, S. König, T. Kono, R. Konoplich, N. Konstantinidis, R. Kopeliansky, S. Koperny, L. Köpke, A. K. Kopp, K. Korcyl, K. Kordas, A. Korn, A. A. Korol, I. Korolkov, E. V. Korolkova, V. A. Korotkov, O. Kortner, S. Kortner, V. V. Kostyukhin, V. M. Kotov, A. Kotwal, C. Kourkoumelis, V. Kouskoura, A. Koutsman, R. Kowalewski, T. Z. Kowalski, W. Kozanecki, A. S. Kozhin, V. Kral, V. A. Kramarenko, G. Kramberger, D. Krasnopevtsev, M. W. Krasny, A. Krasznahorkay, J. K. Kraus, A. Kravchenko, S. Kreiss, M. Kretz, J. Kretzschmar, K. Kreutzfeldt, P. Krieger, K. Kroeninger, H. Kroha, J. Kroll, J. Kroseberg, J. Krstic, U. Kruchonak, H. Krüger, T. Kruker, N. Krumnack, Z. V. Krumshteyn, A. Kruse, M. C. Kruse, M. Kruskal, T. Kubota, S. Kuday, S. Kuehn, A. Kugel, A. Kuhl, T. Kuhl, V. Kukhtin, Y. Kulchitsky, S. Kuleshov, M. Kuna, J. Kunkle, A. Kupco, H. Kurashige, Y. A. Kurochkin, R. Kurumida, V. Kus, E. S. Kuwertz, M. Kuze, J. Kvita, A. La Rosa, L. La Rotonda, C. Lacasta, F. Lacava, J. Lacey, H. Lacker, D. Lacour, V. R. Lacuesta, E. Ladygin, R. Lafaye, B. Laforge, T. Lagouri, S. Lai, H. Laier, L. Lambourne, S. Lammers, C. L. Lampen, W. Lampl, E. Lançon, U. Landgraf, M. P. J. Landon, V. S. Lang, A. J. Lankford, F. Lanni, K. Lantzsch, S. Laplace, C. Lapoire, J. F. Laporte, T. Lari, F. Lasagni Manghi, M. Lassnig, P. Laurelli, W. Lavrijsen, A. T. Law, P. Laycock, O. Le Dortz, E. Le Guirriec, E. Le Menedeu, T. LeCompte, F. Ledroit-Guillon, C. A. Lee, H. Lee, J. S. H. Lee, S. C. Lee, L. Lee, G. Lefebvre, M. Lefebvre, F. Legger, C. Leggett, A. Lehan, M. Lehmacher, G. Lehmann Miotto, X. Lei, W. A. Leight, A. Leisos, A. G. Leister, M. A. L. Leite, R. Leitner, D. Lellouch, B. Lemmer, K. J. C. Leney, T. Lenz, G. Lenzen, B. Lenzi, R. Leone, S. Leone, C. Leonidopoulos, S. Leontsinis, C. Leroy, C. G. Lester, C. M. Lester, M. Levchenko, J. Levêque, D. Levin, L. J. Levinson, M. Levy, A. Lewis, G. H. Lewis, A. M. Leyko, M. Leyton, B. Li, B. Li, H. Li, H. L. Li, L. Li, L. Li, S. Li, Y. Li, Z. Liang, H. Liao, B. Liberti, P. Lichard, K. Lie, J. Liebal, W. Liebig, C. Limbach, A. Limosani, S. C. Lin, T. H. Lin, F. Linde, B. E. Lindquist, J. T. Linnemann, E. Lipeles, A. Lipniacka, M. Lisovyi, T. M. Liss, D. Lissauer, A. Lister, A. M. Litke, B. Liu, D. Liu, J. B. Liu, K. Liu, L. Liu, M. Liu, M. Liu, Y. Liu, M. Livan, S. S. A. Livermore, A. Lleres, J. Llorente Merino, S. L. Lloyd, F. Lo Sterzo, E. Lobodzinska, P. Loch, W. S. Lockman, T. Loddenkoetter, F. K. Loebinger, A. E. Loevschall-Jensen, A. Loginov, T. Lohse, K. Lohwasser, M. Lokajicek, V. P. Lombardo, B. A. Long, J. D. Long, R. E. Long, L. Lopes, D. Lopez Mateos, B. Lopez Paredes, I. Lopez Paz, J. Lorenz, N. Lorenzo Martinez, M. Losada, P. Loscutoff, X. Lou, A. Lounis, J. Love, P. A. Love, A. J. Lowe, F. Lu, N. Lu, H. J. Lubatti, C. Luci, A. Lucotte, F. Luehring, W. Lukas, L. Luminari, O. Lundberg, B. Lund-Jensen, M. Lungwitz, D. Lynn, R. Lysak, E. Lytken, H. Ma, L. L. Ma, G. Maccarrone, A. Macchiolo, J. Machado Miguens, D. Macina, D. Madaffari, R. Madar, H. J. Maddocks, W. F. Mader, A. Madsen, M. Maeno, T. Maeno, A. Maevskiy, E. Magradze, K. Mahboubi, J. Mahlstedt, S. Mahmoud, C. Maiani, C. Maidantchik, A. A. Maier, A. Maio, S. Majewski, Y. Makida, N. Makovec, P. Mal, B. Malaescu, Pa. Malecki, V. P. Maleev, F. Malek, U. Mallik, D. Malon, C. Malone, S. Maltezos, V. M. Malyshev, S. Malyukov, J. Mamuzic, B. Mandelli, L. Mandelli, I. Mandić, R. Mandrysch, J. Maneira, A. Manfredini, L. Manhaes de Andrade Filho, J. A. Manjarres Ramos, A. Mann, P. M. Manning, A. Manousakis-Katsikakis, B. Mansoulie, R. Mantifel, L. Mapelli, L. March, J. F. Marchand, G. Marchiori, M. Marcisovsky, C. P. Marino, M. Marjanovic, C. N. Marques, F. Marroquim, S. P. Marsden, Z. Marshall, L. F. Marti, S. Marti-Garcia, B. Martin, B. Martin, T. A. Martin, V. J. Martin, B. Martin dit Latour, H. Martinez, M. Martinez, S. Martin-Haugh, A. C. Martyniuk, M. Marx, F. Marzano, A. Marzin, L. Masetti, T. Mashimo, R. Mashinistov, J. Masik, A. L. Maslennikov, I. Massa, L. Massa, N. Massol, P. Mastrandrea, A. Mastroberardino, T. Masubuchi, P. Mättig, J. Mattmann, J. Maurer, S. J. Maxfield, D. A. Maximov, R. Mazini, L. Mazzaferro, G. Mc Goldrick, S. P. Mc Kee, A. McCarn, R. L. McCarthy, T. G. McCarthy, N. A. McCubbin, K. W. McFarlane, J. A. Mcfayden, G. Mchedlidze, S. J. McMahon, R. A. McPherson, J. Mechnich, M. Medinnis, S. Meehan, S. Mehlhase, A. Mehta, K. Meier, C. Meineck, B. Meirose, C. Melachrinos, B. R. Mellado Garcia, F. Meloni, A. Mengarelli, S. Menke, E. Meoni, K. M. Mercurio, S. Mergelmeyer, N. Meric, P. Mermod, L. Merola, C. Meroni, F. S. Merritt, H. Merritt, A. Messina, J. Metcalfe, A. S. Mete, C. Meyer, C. Meyer, J.-P. Meyer, J. Meyer, R. P. Middleton, S. Migas, L. Mijović, G. Mikenberg, M. Mikestikova, M. Mikuž, A. Milic, D. W. Miller, C. Mills, A. Milov, D. A. Milstead, D. Milstein, A. A. Minaenko, I. A. Minashvili, A. I. Mincer, B. Mindur, M. Mineev, Y. Ming, L. M. Mir, G. Mirabelli, T. Mitani, J. Mitrevski, V. A. Mitsou, S. Mitsui, A. Miucci, P. S. Miyagawa, J. U. Mjörnmark, T. Moa, K. Mochizuki, S. Mohapatra, W. Mohr, S. Molander, R. Moles-Valls, K. Mönig, C. Monini, J. Monk, E. Monnier, J. Montejo Berlingen, F. Monticelli, S. Monzani, R. W. Moore, N. Morange, D. Moreno, M. Moreno Llácer, P. Morettini, M. Morgenstern, M. Morii, S. Moritz, A. K. Morley, G. Mornacchi, J. D. Morris, L. Morvaj, H. G. Moser, M. Mosidze, J. Moss, K. Motohashi, R. Mount, E. Mountricha, S. V. Mouraviev, E. J. W. Moyse, S. Muanza, R. D. Mudd, F. Mueller, J. Mueller, K. Mueller, T. Mueller, T. Mueller, D. Muenstermann, Y. Munwes, J. A. Murillo Quijada, W. J. Murray, H. Musheghyan, E. Musto, A. G. Myagkov, M. Myska, O. Nackenhorst, J. Nadal, K. Nagai, R. Nagai, Y. Nagai, K. Nagano, A. Nagarkar, Y. Nagasaka, M. Nagel, A. M. Nairz, Y. Nakahama, K. Nakamura, T. Nakamura, I. Nakano, H. Namasivayam, G. Nanava, R. Narayan, T. Nattermann, T. Naumann, G. Navarro, R. Nayyar, H. A. Neal, P. Yu. Nechaeva, T. J. Neep, P. D. Nef, A. Negri, G. Negri, M. Negrini, S. Nektarijevic, C. Nellist, A. Nelson, T. K. Nelson, S. Nemecek, P. Nemethy, A. A. Nepomuceno, M. Nessi, M. S. Neubauer, M. Neumann, R. M. Neves, P. Nevski, P. R. Newman, D. H. Nguyen, R. B. Nickerson, R. Nicolaidou, B. Nicquevert, J. Nielsen, N. Nikiforou, A. Nikiforov, V. Nikolaenko, I. Nikolic-Audit, K. Nikolics, K. Nikolopoulos, P. Nilsson, Y. Ninomiya, A. Nisati, R. Nisius, T. Nobe, L. Nodulman, M. Nomachi, I. Nomidis, S. Norberg, M. Nordberg, O. Novgorodova, S. Nowak, M. Nozaki, L. Nozka, K. Ntekas, G. Nunes Hanninger, T. Nunnemann, E. Nurse, F. Nuti, B. J. O’Brien, F. O’grady, D. C. O’Neil, V. O’Shea, F. G. Oakham, H. Oberlack, T. Obermann, J. Ocariz, A. Ochi, M. I. Ochoa, S. Oda, S. Odaka, H. Ogren, A. Oh, S. H. Oh, C. C. Ohm, H. Ohman, W. Okamura, H. Okawa, Y. Okumura, T. Okuyama, A. Olariu, A. G. Olchevski, S. A. Olivares Pino, D. Oliveira Damazio, E. Oliver Garcia, A. Olszewski, J. Olszowska, A. Onofre, P. U. E. Onyisi, C. J. Oram, M. J. Oreglia, Y. Oren, D. Orestano, N. Orlando, C. Oropeza Barrera, R. S. Orr, B. Osculati, R. Ospanov, G. Otero y Garzon, H. Otono, M. Ouchrif, E. A. Ouellette, F. Ould-Saada, A. Ouraou, K. P. Oussoren, Q. Ouyang, A. Ovcharova, M. Owen, V. E. Ozcan, N. Ozturk, K. Pachal, A. Pacheco Pages, C. Padilla Aranda, M. Pagáčová, S. Pagan Griso, E. Paganis, C. Pahl, F. Paige, P. Pais, K. Pajchel, G. Palacino, S. Palestini, M. Palka, D. Pallin, A. Palma, J. D. Palmer, Y. B. Pan, E. Panagiotopoulou, J. G. Panduro Vazquez, P. Pani, N. Panikashvili, S. Panitkin, D. Pantea, L. Paolozzi, Th. D. Papadopoulou, K. Papageorgiou, A. Paramonov, D. Paredes Hernandez, M. A. Parker, F. Parodi, J. A. Parsons, U. Parzefall, E. Pasqualucci, S. Passaggio, A. Passeri, F. Pastore, Fr. Pastore, G. Pásztor, S. Pataraia, N. D. Patel, J. R. Pater, S. Patricelli, T. Pauly, J. Pearce, L. E. Pedersen, M. Pedersen, S. Pedraza Lopez, R. Pedro, S. V. Peleganchuk, D. Pelikan, H. Peng, B. Penning, J. Penwell, D. V. Perepelitsa, E. Perez Codina, M. T. Pérez García-Estañ, V. Perez Reale, L. Perini, H. Pernegger, S. Perrella, R. Perrino, R. Peschke, V. D. Peshekhonov, K. Peters, R. F. Y. Peters, B. A. Petersen, T. C. Petersen, E. Petit, A. Petridis, C. Petridou, E. Petrolo, F. Petrucci, N. E. Pettersson, R. Pezoa, P. W. Phillips, G. Piacquadio, E. Pianori, A. Picazio, E. Piccaro, M. Piccinini, R. Piegaia, D. T. Pignotti, J. E. Pilcher, A. D. Pilkington, J. Pina, M. Pinamonti, A. Pinder, J. L. Pinfold, A. Pingel, B. Pinto, S. Pires, M. Pitt, C. Pizio, L. Plazak, M.-A. Pleier, V. Pleskot, E. Plotnikova, P. Plucinski, S. Poddar, F. Podlyski, R. Poettgen, L. Poggioli, D. Pohl, M. Pohl, G. Polesello, A. Policicchio, R. Polifka, A. Polini, C. S. Pollard, V. Polychronakos, K. Pommès, L. Pontecorvo, B. G. Pope, G. A. Popeneciu, D. S. Popovic, A. Poppleton, X. Portell Bueso, S. Pospisil, K. Potamianos, I. N. Potrap, C. J. Potter, C. T. Potter, G. Poulard, J. Poveda, V. Pozdnyakov, P. Pralavorio, A. Pranko, S. Prasad, R. Pravahan, S. Prell, D. Price, J. Price, L. E. Price, D. Prieur, M. Primavera, M. Proissl, K. Prokofiev, F. Prokoshin, E. Protopapadaki, S. Protopopescu, J. Proudfoot, M. Przybycien, H. Przysiezniak, E. Ptacek, D. Puddu, E. Pueschel, D. Puldon, M. Purohit, P. Puzo, J. Qian, G. Qin, Y. Qin, A. Quadt, D. R. Quarrie, W. B. Quayle, M. Queitsch-Maitland, D. Quilty, A. Qureshi, V. Radeka, V. Radescu, S. K. Radhakrishnan, P. Radloff, P. Rados, F. Ragusa, G. Rahal, S. Rajagopalan, M. Rammensee, A. S. Randle-Conde, C. Rangel-Smith, K. Rao, F. Rauscher, T. C. Rave, T. Ravenscroft, M. Raymond, A. L. Read, N. P. Readioff, D. M. Rebuzzi, A. Redelbach, G. Redlinger, R. Reece, K. Reeves, L. Rehnisch, H. Reisin, M. Relich, C. Rembser, H. Ren, Z. L. Ren, A. Renaud, M. Rescigno, S. Resconi, O. L. Rezanova, P. Reznicek, R. Rezvani, R. Richter, M. Ridel, P. Rieck, J. Rieger, M. Rijssenbeek, A. Rimoldi, L. Rinaldi, E. Ritsch, I. Riu, F. Rizatdinova, E. Rizvi, S. H. Robertson, A. Robichaud-Veronneau, D. Robinson, J. E. M. Robinson, A. Robson, C. Roda, L. Rodrigues, S. Roe, O. Røhne, S. Rolli, A. Romaniouk, M. Romano, E. Romero Adam, N. Rompotis, M. Ronzani, L. Roos, E. Ros, S. Rosati, K. Rosbach, M. Rose, P. Rose, P. L. Rosendahl, O. Rosenthal, V. Rossetti, E. Rossi, L. P. Rossi, R. Rosten, M. Rotaru, I. Roth, J. Rothberg, D. Rousseau, C. R. Royon, A. Rozanov, Y. Rozen, X. Ruan, F. Rubbo, I. Rubinskiy, V. I. Rud, C. Rudolph, M. S. Rudolph, F. Rühr, A. Ruiz-Martinez, Z. Rurikova, N. A. Rusakovich, A. Ruschke, J. P. Rutherfoord, N. Ruthmann, Y. F. Ryabov, M. Rybar, G. Rybkin, N. C. Ryder, A. F. Saavedra, S. Sacerdoti, A. Saddique, I. Sadeh, H. F.-W. Sadrozinski, R. Sadykov, F. Safai Tehrani, H. Sakamoto, Y. Sakurai, G. Salamanna, A. Salamon, M. Saleem, D. Salek, P. H. Sales De Bruin, D. Salihagic, A. Salnikov, J. Salt, D. Salvatore, F. Salvatore, A. Salvucci, A. Salzburger, D. Sampsonidis, A. Sanchez, J. Sánchez, V. Sanchez Martinez, H. Sandaker, R. L. Sandbach, H. G. Sander, M. P. Sanders, M. Sandhoff, T. Sandoval, C. Sandoval, R. Sandstroem, D. P. C. Sankey, A. Sansoni, C. Santoni, R. Santonico, H. Santos, I. Santoyo Castillo, K. Sapp, A. Sapronov, J. G. Saraiva, B. Sarrazin, G. Sartisohn, O. Sasaki, Y. Sasaki, G. Sauvage, E. Sauvan, P. Savard, D. O. Savu, C. Sawyer, L. Sawyer, D. H. Saxon, J. Saxon, C. Sbarra, A. Sbrizzi, T. Scanlon, D. A. Scannicchio, M. Scarcella, V. Scarfone, J. Schaarschmidt, P. Schacht, D. Schaefer, R. Schaefer, S. Schaepe, S. Schaetzel, U. Schäfer, A. C. Schaffer, D. Schaile, R. D. Schamberger, V. Scharf, V. A. Schegelsky, D. Scheirich, M. Schernau, M. I. Scherzer, C. Schiavi, J. Schieck, C. Schillo, M. Schioppa, S. Schlenker, E. Schmidt, K. Schmieden, C. Schmitt, S. Schmitt, B. Schneider, Y. J. Schnellbach, U. Schnoor, L. Schoeffel, A. Schoening, B. D. Schoenrock, A. L. S. Schorlemmer, M. Schott, D. Schouten, J. Schovancova, S. Schramm, M. Schreyer, C. Schroeder, N. Schuh, M. J. Schultens, H.-C. Schultz-Coulon, H. Schulz, M. Schumacher, B. A. Schumm, Ph. Schune, C. Schwanenberger, A. Schwartzman, T. A. Schwarz, Ph. Schwegler, Ph. Schwemling, R. Schwienhorst, J. Schwindling, T. Schwindt, M. Schwoerer, F. G. Sciacca, E. Scifo, G. Sciolla, W. G. Scott, F. Scuri, F. Scutti, J. Searcy, G. Sedov, E. Sedykh, S. C. Seidel, A. Seiden, F. Seifert, J. M. Seixas, G. Sekhniaidze, S. J. Sekula, K. E. Selbach, D. M. Seliverstov, G. Sellers, N. Semprini-Cesari, C. Serfon, L. Serin, L. Serkin, T. Serre, R. Seuster, H. Severini, T. Sfiligoj, F. Sforza, A. Sfyrla, E. Shabalina, M. Shamim, L. Y. Shan, R. Shang, J. T. Shank, M. Shapiro, P. B. Shatalov, K. Shaw, C. Y. Shehu, P. Sherwood, L. Shi, S. Shimizu, C. O. Shimmin, M. Shimojima, M. Shiyakova, A. Shmeleva, M. J. Shochet, D. Short, S. Shrestha, E. Shulga, M. A. Shupe, S. Shushkevich, P. Sicho, O. Sidiropoulou, D. Sidorov, A. Sidoti, F. Siegert, Dj. Sijacki, J. Silva, Y. Silver, D. Silverstein, S. B. Silverstein, V. Simak, O. Simard, Lj. Simic, S. Simion, E. Simioni, B. Simmons, R. Simoniello, M. Simonyan, P. Sinervo, N. B. Sinev, V. Sipica, G. Siragusa, A. Sircar, A. N. Sisakyan, S. Yu. Sivoklokov, J. Sjölin, T. B. Sjursen, H. P. Skottowe, K. Yu. Skovpen, P. Skubic, M. Slater, T. Slavicek, K. Sliwa, V. Smakhtin, B. H. Smart, L. Smestad, S. Yu. Smirnov, Y. Smirnov, L. N. Smirnova, O. Smirnova, K. M. Smith, M. Smizanska, K. Smolek, A. A. Snesarev, G. Snidero, S. Snyder, R. Sobie, F. Socher, A. Soffer, D. A. Soh, C. A. Solans, M. Solar, J. Solc, E. Yu. Soldatov, U. Soldevila, A. A. Solodkov, A. Soloshenko, O. V. Solovyanov, V. Solovyev, P. Sommer, H. Y. Song, N. Soni, A. Sood, A. Sopczak, B. Sopko, V. Sopko, V. Sorin, M. Sosebee, R. Soualah, P. Soueid, A. M. Soukharev, D. South, S. Spagnolo, F. Spanò, W. R. Spearman, F. Spettel, R. Spighi, G. Spigo, L. A. Spiller, M. Spousta, T. Spreitzer, B. Spurlock, R. D. St. Denis, S. Staerz, J. Stahlman, R. Stamen, S. Stamm, E. Stanecka, R. W. Stanek, C. Stanescu, M. Stanescu-Bellu, M. M. Stanitzki, S. Stapnes, E. A. Starchenko, J. Stark, P. Staroba, P. Starovoitov, R. Staszewski, P. Stavina, P. Steinberg, B. Stelzer, H. J. Stelzer, O. Stelzer-Chilton, H. Stenzel, S. Stern, G. A. Stewart, J. A. Stillings, M. C. Stockton, M. Stoebe, G. Stoicea, P. Stolte, S. Stonjek, A. R. Stradling, A. Straessner, M. E. Stramaglia, J. Strandberg, S. Strandberg, A. Strandlie, E. Strauss, M. Strauss, P. Strizenec, R. Ströhmer, D. M. Strom, R. Stroynowski, A. Strubig, S. A. Stucci, B. Stugu, N. A. Styles, D. Su, J. Su, R. Subramaniam, A. Succurro, Y. Sugaya, C. Suhr, M. Suk, V. V. Sulin, S. Sultansoy, T. Sumida, S. Sun, X. Sun, J. E. Sundermann, K. Suruliz, G. Susinno, M. R. Sutton, Y. Suzuki, M. Svatos, S. Swedish, M. Swiatlowski, I. Sykora, T. Sykora, D. Ta, C. Taccini, K. Tackmann, J. Taenzer, A. Taffard, R. Tafirout, N. Taiblum, H. Takai, R. Takashima, H. Takeda, T. Takeshita, Y. Takubo, M. Talby, A. A. Talyshev, J. Y. C. Tam, K. G. Tan, J. Tanaka, R. Tanaka, S. Tanaka, S. Tanaka, A. J. Tanasijczuk, B. B. Tannenwald, N. Tannoury, S. Tapprogge, S. Tarem, F. Tarrade, G. F. Tartarelli, P. Tas, M. Tasevsky, T. Tashiro, E. Tassi, A. Tavares Delgado, Y. Tayalati, F. E. Taylor, G. N. Taylor, W. Taylor, F. A. Teischinger, M. Teixeira Dias Castanheira, P. Teixeira-Dias, K. K. Temming, H. Ten Kate, P. K. Teng, J. J. Teoh, S. Terada, K. Terashi, J. Terron, S. Terzo, M. Testa, R. J. Teuscher, J. Therhaag, T. Theveneaux-Pelzer, J. P. Thomas, J. Thomas-Wilsker, E. N. Thompson, P. D. Thompson, P. D. Thompson, R. J. Thompson, A. S. Thompson, L. A. Thomsen, E. Thomson, M. Thomson, W. M. Thong, R. P. Thun, F. Tian, M. J. Tibbetts, V. O. Tikhomirov, Yu. A. Tikhonov, S. Timoshenko, E. Tiouchichine, P. Tipton, S. Tisserant, T. Todorov, S. Todorova-Nova, B. Toggerson, J. Tojo, S. Tokár, K. Tokushuku, K. Tollefson, E. Tolley, L. Tomlinson, M. Tomoto, L. Tompkins, K. Toms, N. D. Topilin, E. Torrence, H. Torres, E. Torró Pastor, J. Toth, F. Touchard, D. R. Tovey, H. L. Tran, T. Trefzger, L. Tremblet, A. Tricoli, I. M. Trigger, S. Trincaz-Duvoid, M. F. Tripiana, W. Trischuk, B. Trocmé, C. Troncon, M. Trottier-McDonald, M. Trovatelli, P. True, M. Trzebinski, A. Trzupek, C. Tsarouchas, J. C.-L. Tseng, P. V. Tsiareshka, D. Tsionou, G. Tsipolitis, N. Tsirintanis, S. Tsiskaridze, V. Tsiskaridze, E. G. Tskhadadze, I. I. Tsukerman, V. Tsulaia, S. Tsuno, D. Tsybychev, A. Tudorache, V. Tudorache, A. N. Tuna, S. A. Tupputi, S. Turchikhin, D. Turecek, I. Turk Cakir, R. Turra, P. M. Tuts, A. Tykhonov, M. Tylmad, M. Tyndel, K. Uchida, I. Ueda, R. Ueno, M. Ughetto, M. Ugland, M. Uhlenbrock, F. Ukegawa, G. Unal, A. Undrus, G. Unel, F. C. Ungaro, Y. Unno, C. Unverdorben, D. Urbaniec, P. Urquijo, G. Usai, A. Usanova, L. Vacavant, V. Vacek, B. Vachon, N. Valencic, S. Valentinetti, A. Valero, L. Valery, S. Valkar, E. Valladolid Gallego, S. Vallecorsa, J. A. Valls Ferrer, W. Van Den Wollenberg, P. C. Van Der Deijl, R. van der Geer, H. van der Graaf, R. Van Der Leeuw, D. van der Ster, N. van Eldik, P. van Gemmeren, J. Van Nieuwkoop, I. van Vulpen, M. C. van Woerden, M. Vanadia, W. Vandelli, R. Vanguri, A. Vaniachine, P. Vankov, F. Vannucci, G. Vardanyan, R. Vari, E. W. Varnes, T. Varol, D. Varouchas, A. Vartapetian, K. E. Varvell, F. Vazeille, T. Vazquez Schroeder, J. Veatch, F. Veloso, S. Veneziano, A. Ventura, D. Ventura, M. Venturi, N. Venturi, A. Venturini, V. Vercesi, M. Verducci, W. Verkerke, J. C. Vermeulen, A. Vest, M. C. Vetterli, O. Viazlo, I. Vichou, T. Vickey, O. E. Vickey Boeriu, G. H. A. Viehhauser, S. Viel, R. Vigne, M. Villa, M. Villaplana Perez, E. Vilucchi, M. G. Vincter, V. B. Vinogradov, J. Virzi, I. Vivarelli, F. Vives Vaque, S. Vlachos, D. Vladoiu, M. Vlasak, A. Vogel, M. Vogel, P. Vokac, G. Volpi, M. Volpi, H. von der Schmitt, H. von Radziewski, E. von Toerne, V. Vorobel, K. Vorobev, M. Vos, R. Voss, J. H. Vossebeld, N. Vranjes, M. Vranjes Milosavljevic, V. Vrba, M. Vreeswijk, T. Vu Anh, R. Vuillermet, I. Vukotic, Z. Vykydal, P. Wagner, W. Wagner, H. Wahlberg, S. Wahrmund, J. Wakabayashi, J. Walder, R. Walker, W. Walkowiak, R. Wall, P. Waller, B. Walsh, C. Wang, C. Wang, F. Wang, H. Wang, H. Wang, J. Wang, J. Wang, K. Wang, R. Wang, S. M. Wang, T. Wang, X. Wang, C. Wanotayaroj, A. Warburton, C. P. Ward, D. R. Wardrope, M. Warsinsky, A. Washbrook, C. Wasicki, P. M. Watkins, A. T. Watson, I. J. Watson, M. F. Watson, G. Watts, S. Watts, B. M. Waugh, S. Webb, M. S. Weber, S. W. Weber, J. S. Webster, A. R. Weidberg, P. Weigell, B. Weinert, J. Weingarten, C. Weiser, H. Weits, P. S. Wells, T. Wenaus, D. Wendland, Z. Weng, T. Wengler, S. Wenig, N. Wermes, M. Werner, P. Werner, M. Wessels, J. Wetter, K. Whalen, A. White, M. J. White, R. White, S. White, D. Whiteson, D. Wicke, F. J. Wickens, W. Wiedenmann, M. Wielers, P. Wienemann, C. Wiglesworth, L. A. M. Wiik-Fuchs, P. A. Wijeratne, A. Wildauer, M. A. Wildt, H. G. Wilkens, J. Z. Will, H. H. Williams, S. Williams, C. Willis, S. Willocq, A. Wilson, J. A. Wilson, I. Wingerter-Seez, F. Winklmeier, B. T. Winter, M. Wittgen, T. Wittig, J. Wittkowski, S. J. Wollstadt, M. W. Wolter, H. Wolters, B. K. Wosiek, J. Wotschack, M. J. Woudstra, K. W. Wozniak, M. Wright, M. Wu, S. L. Wu, X. Wu, Y. Wu, E. Wulf, T. R. Wyatt, B. M. Wynne, S. Xella, M. Xiao, D. Xu, L. Xu, B. Yabsley, S. Yacoob, R. Yakabe, M. Yamada, H. Yamaguchi, Y. Yamaguchi, A. Yamamoto, K. Yamamoto, S. Yamamoto, T. Yamamura, T. Yamanaka, K. Yamauchi, Y. Yamazaki, Z. Yan, H. Yang, H. Yang, U. K. Yang, Y. Yang, S. Yanush, L. Yao, W.-M. Yao, Y. Yasu, E. Yatsenko, K. H. Yau Wong, J. Ye, S. Ye, I. Yeletskikh, A. L. Yen, E. Yildirim, M. Yilmaz, R. Yoosoofmiya, K. Yorita, R. Yoshida, K. Yoshihara, C. Young, C. J. S. Young, S. Youssef, D. R. Yu, J. Yu, J. M. Yu, J. Yu, L. Yuan, A. Yurkewicz, I. Yusuff, B. Zabinski, R. Zaidan, A. M. Zaitsev, A. Zaman, S. Zambito, L. Zanello, D. Zanzi, C. Zeitnitz, M. Zeman, A. Zemla, K. Zengel, O. Zenin, T. Ženiš, D. Zerwas, G. Zevi della Porta, D. Zhang, F. Zhang, H. Zhang, J. Zhang, L. Zhang, X. Zhang, Z. Zhang, Z. Zhao, A. Zhemchugov, J. Zhong, B. Zhou, L. Zhou, N. Zhou, C. G. Zhu, H. Zhu, J. Zhu, Y. Zhu, X. Zhuang, K. Zhukov, A. Zibell, D. Zieminska, N. I. Zimine, C. Zimmermann, R. Zimmermann, S. Zimmermann, S. Zimmermann, Z. Zinonos, M. Ziolkowski, G. Zobernig, A. Zoccoli, M. zur Nedden, G. Zurzolo, V. Zutshi, L. Zwalinski

**Affiliations:** 1 Department of Physics, University of Adelaide, Adelaide, Australia; 2 Physics Department, SUNY Albany, Albany, NY USA; 3 Department of Physics, University of Alberta, Edmonton, AB Canada; 4 Department of Physics, Ankara University, Ankara, Turkey; Department of Physics, Gazi University, Ankara, Turkey; Division of Physics, TOBB University of Economics and Technology, Ankara, Turkey; Turkish Atomic Energy Authority, Ankara, Turkey; 5 LAPP, CNRS/IN2P3 and Université de Savoie, Annecy-le-Vieux, France; 6 High Energy Physics Division, Argonne National Laboratory, Argonne, IL USA; 7 Department of Physics, University of Arizona, Tucson, AZ USA; 8 Department of Physics, The University of Texas at Arlington, Arlington, TX USA; 9 Physics Department, University of Athens, Athens, Greece; 10 Physics Department, National Technical University of Athens, Zografou, Greece; 11 Institute of Physics, Azerbaijan Academy of Sciences, Baku, Azerbaijan; 12 Institut de Física d’Altes Energies and Departament de Física de la Universitat Autònoma de Barcelona, Barcelona, Spain; 13 Institute of Physics, University of Belgrade, Belgrade, Serbia; Vinca Institute of Nuclear Sciences, University of Belgrade, Belgrade, Serbia; 14 Department for Physics and Technology, University of Bergen, Bergen, Norway; 15 Physics Division, Lawrence Berkeley National Laboratory and University of California, Berkeley, CA USA; 16 Department of Physics, Humboldt University, Berlin, Germany; 17 Albert Einstein Center for Fundamental Physics and Laboratory for High Energy Physics, University of Bern, Bern, Switzerland; 18 School of Physics and Astronomy, University of Birmingham, Birmingham, UK; 19 Department of Physics, Bogazici University, Istanbul, Turkey; Department of Physics, Dogus University, Istanbul, Turkey; Department of Physics Engineering, Gaziantep University, Gaziantep, Turkey; 20 INFN Sezione di Bologna, Bologna, Italy; Dipartimento di Fisica e Astronomia, Università di Bologna, Bologna, Italy; 21 Physikalisches Institut, University of Bonn, Bonn, Germany; 22 Department of Physics, Boston University, Boston, MA USA; 23 Department of Physics, Brandeis University, Waltham, MA USA; 24 Universidade Federal do Rio De Janeiro COPPE/EE/IF, Rio de Janeiro, Brazil; Federal University of Juiz de Fora (UFJF), Juiz de Fora, Brazil; Federal University of Sao Joao del Rei (UFSJ), Sao Joao del Rei, Brazil; Instituto de Fisica, Universidade de Sao Paulo, São Paulo, Brazil; 25 Physics Department, Brookhaven National Laboratory, Upton, NY USA; 26 National Institute of Physics and Nuclear Engineering, Bucharest, Romania; Physics Department, National Institute for Research and Development of Isotopic and Molecular Technologies, Cluj Napoca, Romania; University Politehnica Bucharest, Bucharest, Romania; West University in Timisoara, Timisoara, Romania; 27 Departamento de Física, Universidad de Buenos Aires, Buenos Aires, Argentina; 28 Cavendish Laboratory, University of Cambridge, Cambridge, UK; 29 Department of Physics, Carleton University, Ottawa, ON Canada; 30 CERN, Geneva, Switzerland; 31 Enrico Fermi Institute, University of Chicago, Chicago, IL USA; 32 Departamento de Física, Pontificia Universidad Católica de Chile, Santiago, Chile; Departamento de Física, Universidad Técnica Federico Santa María, Valparaiso, Chile; 33 Institute of High Energy Physics, Chinese Academy of Sciences, Beijing, China; Department of Modern Physics, University of Science and Technology of China, Hefei, Anhui, China; Department of Physics, Nanjing University, Nanjing, Jiangsu, China; School of Physics, Shandong University, Jinan, Shandong, China; Physics Department, Shanghai Jiao Tong University, Shanghai, China; 34 Laboratoire de Physique Corpusculaire, Clermont Université and Université Blaise Pascal and CNRS/IN2P3, Clermont-Ferrand, France; 35 Nevis Laboratory, Columbia University, Irvington, NY USA; 36 Niels Bohr Institute, University of Copenhagen, Copenhagen, Denmark; 37 INFN Gruppo Collegato di Cosenza, Laboratori Nazionali di Frascati, Frascati, Italy; Dipartimento di Fisica, Università della Calabria, Rende, Italy; 38 Faculty of Physics and Applied Computer Science, AGH University of Science and Technology, Kraków, Poland; Marian Smoluchowski Institute of Physics, Jagiellonian University, Kraków, Poland; 39 The Henryk Niewodniczanski Institute of Nuclear Physics, Polish Academy of Sciences, Kraków, Poland; 40 Physics Department, Southern Methodist University, Dallas, TX USA; 41 Physics Department, University of Texas at Dallas, Richardson, TX USA; 42 DESY, Hamburg and Zeuthen, Germany; 43 Institut für Experimentelle Physik IV, Technische Universität Dortmund, Dortmund, Germany; 44 Institut für Kern- und Teilchenphysik, Technische Universität Dresden, Dresden, Germany; 45 Department of Physics, Duke University, Durham, NC USA; 46 SUPA-School of Physics and Astronomy, University of Edinburgh, Edinburgh, UK; 47 INFN Laboratori Nazionali di Frascati, Frascati, Italy; 48 Fakultät für Mathematik und Physik, Albert-Ludwigs-Universität, Freiburg, Germany; 49 Section de Physique, Université de Genève, Geneva, Switzerland; 50 INFN Sezione di Genova, Genoa, Italy; Dipartimento di Fisica, Università di Genova, Genoa, Italy; 51 E. Andronikashvili Institute of Physics, Iv. Javakhishvili Tbilisi State University, Tbilisi, Georgia; High Energy Physics Institute, Tbilisi State University, Tbilisi, Georgia; 52 II Physikalisches Institut, Justus-Liebig-Universität Giessen, Giessen, Germany; 53 SUPA-School of Physics and Astronomy, University of Glasgow, Glasgow, UK; 54 II Physikalisches Institut, Georg-August-Universität, Göttingen, Germany; 55 Laboratoire de Physique Subatomique et de Cosmologie, Université Grenoble-Alpes, CNRS/IN2P3, Grenoble, France; 56 Department of Physics, Hampton University, Hampton, VA USA; 57 Laboratory for Particle Physics and Cosmology, Harvard University, Cambridge, MA USA; 58 Kirchhoff-Institut für Physik, Ruprecht-Karls-Universität Heidelberg, Heidelberg, Germany; Physikalisches Institut, Ruprecht-Karls-Universität Heidelberg, Heidelberg, Germany; ZITI Institut für technische Informatik, Ruprecht-Karls-Universität Heidelberg, Mannheim, Germany; 59 Faculty of Applied Information Science, Hiroshima Institute of Technology, Hiroshima, Japan; 60 Department of Physics, Indiana University, Bloomington, IN USA; 61 Institut für Astro- und Teilchenphysik, Leopold-Franzens-Universität, Innsbruck, Austria; 62 University of Iowa, Iowa City, IA USA; 63 Department of Physics and Astronomy, Iowa State University, Ames, IA USA; 64 Joint Institute for Nuclear Research, JINR Dubna, Dubna, Russia; 65 KEK, High Energy Accelerator Research Organization, Tsukuba, Japan; 66 Graduate School of Science, Kobe University, Kobe, Japan; 67 Faculty of Science, Kyoto University, Kyoto, Japan; 68 Kyoto University of Education, Kyoto, Japan; 69 Department of Physics, Kyushu University, Fukuoka, Japan; 70 Instituto de Física La Plata, Universidad Nacional de La Plata and CONICET, La Plata, Argentina; 71 Physics Department, Lancaster University, Lancaster, UK; 72 INFN Sezione di Lecce, Lecce, Italy; Dipartimento di Matematica e Fisica, Università del Salento, Lecce, Italy; 73 Oliver Lodge Laboratory, University of Liverpool, Liverpool, UK; 74 Department of Physics, Jožef Stefan Institute and University of Ljubljana, Ljubljana, Slovenia; 75 School of Physics and Astronomy, Queen Mary University of London, London, UK; 76 Department of Physics, Royal Holloway University of London, Surrey, UK; 77 Department of Physics and Astronomy, University College London, London, UK; 78 Louisiana Tech University, Ruston, LA USA; 79 Laboratoire de Physique Nucléaire et de Hautes Energies, UPMC and Université Paris-Diderot and CNRS/IN2P3, Paris, France; 80 Fysiska institutionen, Lunds universitet, Lund, Sweden; 81 Departamento de Fisica Teorica C-15, Universidad Autonoma de Madrid, Madrid, Spain; 82 Institut für Physik, Universität Mainz, Mainz, Germany; 83 School of Physics and Astronomy, University of Manchester, Manchester, UK; 84 CPPM, Aix-Marseille Université and CNRS/IN2P3, Marseille, France; 85 Department of Physics, University of Massachusetts, Amherst, MA USA; 86 Department of Physics, McGill University, Montreal, QC Canada; 87 School of Physics, University of Melbourne, Parkville, VIC Australia; 88 Department of Physics, The University of Michigan, Ann Arbor, MI USA; 89 Department of Physics and Astronomy, Michigan State University, East Lansing, MI USA; 90 INFN Sezione di Milano, Milan, Italy; Dipartimento di Fisica, Università di Milano, Milan, Italy; 91 B.I. Stepanov Institute of Physics, National Academy of Sciences of Belarus, Minsk, Republic of Belarus; 92 National Scientific and Educational Centre for Particle and High Energy Physics, Minsk, Republic of Belarus; 93 Department of Physics, Massachusetts Institute of Technology, Cambridge, MA USA; 94 Group of Particle Physics, University of Montreal, Montreal, QC Canada; 95 P.N. Lebedev Institute of Physics, Academy of Sciences, Moscow, Russia; 96 Institute for Theoretical and Experimental Physics (ITEP), Moscow, Russia; 97 Moscow Engineering and Physics Institute (MEPhI), Moscow, Russia; 98 D.V. Skobeltsyn Institute of Nuclear Physics, M.V. Lomonosov Moscow State University, Moscow, Russia; 99 Fakultät für Physik, Ludwig-Maximilians-Universität München, Munich, Germany; 100 Max-Planck-Institut für Physik (Werner-Heisenberg-Institut), Munich, Germany; 101 Nagasaki Institute of Applied Science, Nagasaki, Japan; 102 Graduate School of Science and Kobayashi-Maskawa Institute, Nagoya University, Nagoya, Japan; 103 INFN Sezione di Napoli, Naples, Italy; Dipartimento di Fisica, Università di Napoli, Naples, Italy; 104 Department of Physics and Astronomy, University of New Mexico, Albuquerque, NM USA; 105 Institute for Mathematics, Astrophysics and Particle Physics, Radboud University Nijmegen/Nikhef, Nijmegen, The Netherlands; 106 Nikhef National Institute for Subatomic Physics and University of Amsterdam, Amsterdam, The Netherlands; 107 Department of Physics, Northern Illinois University, DeKalb, IL USA; 108 Budker Institute of Nuclear Physics, SB RAS, Novosibirsk, Russia; 109 Department of Physics, New York University, New York, NY USA; 110 Ohio State University, Columbus, OH USA; 111 Faculty of Science, Okayama University, Okayama, Japan; 112 Homer L. Dodge Department of Physics and Astronomy, University of Oklahoma, Norman, OK USA; 113 Department of Physics, Oklahoma State University, Stillwater, OK USA; 114 Palacký University, RCPTM, Olomouc, Czech Republic; 115 Center for High Energy Physics, University of Oregon, Eugene, OR USA; 116 LAL, Université Paris-Sud and CNRS/IN2P3, Orsay, France; 117 Graduate School of Science, Osaka University, Osaka, Japan; 118 Department of Physics, University of Oslo, Oslo, Norway; 119 Department of Physics, Oxford University, Oxford, UK; 120 INFN Sezione di Pavia, Pavia, Italy; Dipartimento di Fisica, Università di Pavia, Pavia, Italy; 121 Department of Physics, University of Pennsylvania, Philadelphia, PA USA; 122 Petersburg Nuclear Physics Institute, Gatchina, Russia; 123 INFN Sezione di Pisa, Pisa, Italy; Dipartimento di Fisica E. Fermi, Università di Pisa, Pisa, Italy; 124 Department of Physics and Astronomy, University of Pittsburgh, Pittsburgh, PA USA; 125 Laboratorio de Instrumentacao e Fisica Experimental de Particulas-LIP, Lisbon, Portugal; Faculdade de Ciências, Universidade de Lisboa, Lisbon, Portugal; Department of Physics, University of Coimbra, Coimbra, Portugal; Centro de Física Nuclear da Universidade de Lisboa, Lisbon, Portugal; Departamento de Fisica, Universidade do Minho, Braga, Portugal; Departamento de Fisica Teorica y del Cosmos and CAFPE, Universidad de Granada, Granada, Spain; Dep Fisica and CEFITEC of Faculdade de Ciencias e Tecnologia, Universidade Nova de Lisboa, Caparica, Portugal; 126 Institute of Physics, Academy of Sciences of the Czech Republic, Prague, Czech Republic; 127 Czech Technical University in Prague, Prague, Czech Republic; 128 Faculty of Mathematics and Physics, Charles University in Prague, Prague, Czech Republic; 129 State Research Center Institute for High Energy Physics, Protvino, Russia; 130 Particle Physics Department, Rutherford Appleton Laboratory, Didcot, UK; 131 Physics Department, University of Regina, Regina, SK Canada; 132 Ritsumeikan University, Kusatsu, Shiga Japan; 133 INFN Sezione di Roma, Rome, Italy; Dipartimento di Fisica, Sapienza Università di Roma, Rome, Italy; 134 INFN Sezione di Roma Tor Vergata, Rome, Italy; Dipartimento di Fisica, Università di Roma Tor Vergata, Rome, Italy; 135 INFN Sezione di Roma Tre, Rome, Italy; Dipartimento di Matematica e Fisica, Università Roma Tre, Rome, Italy; 136 Faculté des Sciences Ain Chock, Réseau Universitaire de Physique des Hautes Energies-Université Hassan II, Casablanca, Morocco; Centre National de l’Energie des Sciences Techniques Nucleaires, Rabat, Morocco; Faculté des Sciences Semlalia, Université Cadi Ayyad, LPHEA-Marrakech, Marrakech, Morocco; Faculté des Sciences, Université Mohamed Premier and LPTPM, Oujda, Morocco; Faculté des Sciences, Université Mohammed V-Agdal, Rabat, Morocco; 137 DSM/IRFU (Institut de Recherches sur les Lois Fondamentales de l’Univers), CEA Saclay (Commissariat à l’Energie Atomique et aux Energies Alternatives), Gif-sur-Yvette, France; 138 Santa Cruz Institute for Particle Physics, University of California Santa Cruz, Santa Cruz, CA USA; 139 Department of Physics, University of Washington, Seattle, WA USA; 140 Department of Physics and Astronomy, University of Sheffield, Sheffield, UK; 141 Department of Physics, Shinshu University, Nagano, Japan; 142 Fachbereich Physik, Universität Siegen, Siegen, Germany; 143 Department of Physics, Simon Fraser University, Burnaby, BC Canada; 144 SLAC National Accelerator Laboratory, Stanford, CA USA; 145 Faculty of Mathematics, Physics and Informatics, Comenius University, Bratislava, Slovak Republic; Department of Subnuclear Physics, Institute of Experimental Physics of the Slovak Academy of Sciences, Kosice, Slovak Republic; 146 Department of Physics, University of Cape Town, Cape Town, South Africa; Department of Physics, University of Johannesburg, Johannesburg, South Africa; School of Physics, University of the Witwatersrand, Johannesburg, South Africa; 147 Department of Physics, Stockholm University, Stockholm, Sweden; The Oskar Klein Centre, Stockholm, Sweden; 148 Physics Department, Royal Institute of Technology, Stockholm, Sweden; 149 Departments of Physics and Astronomy and Chemistry, Stony Brook University, Stony Brook, NY USA; 150 Department of Physics and Astronomy, University of Sussex, Brighton, UK; 151 School of Physics, University of Sydney, Sydney, Australia; 152 Institute of Physics, Academia Sinica, Taipei, Taiwan; 153 Department of Physics, Technion: Israel Institute of Technology, Haifa, Israel; 154 Raymond and Beverly Sackler School of Physics and Astronomy, Tel Aviv University, Tel Aviv, Israel; 155 Department of Physics, Aristotle University of Thessaloniki, Thessaloniki, Greece; 156 International Center for Elementary Particle Physics and Department of Physics, The University of Tokyo, Tokyo, Japan; 157 Graduate School of Science and Technology, Tokyo Metropolitan University, Tokyo, Japan; 158 Department of Physics, Tokyo Institute of Technology, Tokyo, Japan; 159 Department of Physics, University of Toronto, Toronto, ON Canada; 160 TRIUMF, Vancouver, BC, Canada; Department of Physics and Astronomy, York University, Toronto, ON Canada; 161 Faculty of Pure and Applied Sciences, University of Tsukuba, Tsukuba, Japan; 162 Department of Physics and Astronomy, Tufts University, Medford, MA USA; 163 Centro de Investigaciones, Universidad Antonio Narino, Bogota, Colombia; 164 Department of Physics and Astronomy, University of California Irvine, Irvine, CA USA; 165 INFN Gruppo Collegato di Udine, Sezione di Trieste, Udine, Italy; ICTP, Trieste, Italy; Dipartimento di Chimica, Fisica e Ambiente, Università di Udine, Udine, Italy; 166 Department of Physics, University of Illinois, Urbana, IL USA; 167 Department of Physics and Astronomy, University of Uppsala, Uppsala, Sweden; 168 Instituto de Física Corpuscular (IFIC) and Departamento de Física Atómica, Molecular y Nuclear and Departamento de Ingeniería Electrónica and Instituto de Microelectrónica de Barcelona (IMB-CNM), University of Valencia and CSIC, Valencia, Spain; 169 Department of Physics, University of British Columbia, Vancouver, BC Canada; 170 Department of Physics and Astronomy, University of Victoria, Victoria, BC Canada; 171 Department of Physics, University of Warwick, Coventry, UK; 172 Waseda University, Tokyo, Japan; 173 Department of Particle Physics, The Weizmann Institute of Science, Rehovot, Israel; 174 Department of Physics, University of Wisconsin, Madison, WI USA; 175 Fakultät für Physik und Astronomie, Julius-Maximilians-Universität, Würzburg, Germany; 176 Fachbereich C Physik, Bergische Universität Wuppertal, Wuppertal, Germany; 177 Department of Physics, Yale University, New Haven, CT USA; 178 Yerevan Physics Institute, Yerevan, Armenia; 179 Centre de Calcul de l’Institut National de Physique Nucléaire et de Physique des Particules (IN2P3), Villeurbanne, France; 180 CERN, 1211 Geneva 23, Switzerland

## Abstract

The ratio of the production cross sections for $W$ and $Z$ bosons in association with jets has been measured in proton–proton collisions at $\sqrt{s}=7\,\mathrm {TeV}$ with the ATLAS experiment at the Large Hadron Collider. The measurement is based on the entire 2011 dataset, corresponding to an integrated luminosity of $4.6\,\mathrm {fb}^{-1}$. Inclusive and differential cross-section ratios for massive vector bosons decaying to electrons and muons are measured in association with jets with transverse momentum $p_\mathrm {T} >30\,\mathrm {GeV}$ and jet rapidity $|y| < 4.4$. The measurements are compared to next-to-leading-order perturbative QCD calculations and to predictions from different Monte Carlo generators implementing leading-order matrix elements supplemented by parton showers.

## Introduction

Precise measurements of the production of vector bosons in association with jets are important tests of quantum chromodynamics (QCD) and provide constraints on background processes to Higgs boson studies and to searches for new physics. The measurement of the ratio of $W\,\mathtt + \,\mathrm {jets}$ to $Z \,\mathtt + \,\mathrm {jets}$ 1 production cross sections, termed $R_{\mathrm {jets}}$, directly probes the difference between the kinematic distributions of the jet system recoiling against the $W$ or $Z$ bosons.

In comparison to separate $W\,\mathtt + \,\mathrm {jets}$ and $Z \,\mathtt + \,\mathrm {jets}$ cross section measurements, the $R_{\mathrm {jets}}$ measurement is a more precise test of perturbative QCD (pQCD), since some experimental uncertainties and effects from non-perturbative processes, such as hadronization and multi-parton interactions, are greatly reduced in the ratio. This allows precise comparisons with state-of-the-art Monte Carlo simulations and next-to-leading-order (NLO) perturbative QCD calculations to be made.

At low energies, the difference in vector-boson masses translates to a change in momentum transfer between incoming partons and thus different hadronic radiation patterns. In addition, the parton distribution functions of the proton (PDFs) imply different quark–gluon and quark–antiquark contributions to $W\,\mathtt + \,\mathrm {jets}$ and $Z \,\mathtt + \,\mathrm {jets}$ processes.

At very high energies, the vector-boson mass difference is not large relative to the momentum transfer, so differences between $W\,\mathtt + \,\mathrm {jets}$ and $Z \,\mathtt + \,\mathrm {jets}$ production are expected to decrease, even though some differences in the parton distribution functions remain. A precise measurement of $R_{\mathrm {jets}}$ can therefore be used, in the context of searches for new particles or interactions beyond the Standard Model, to infer the $W\,\mathtt + \,\mathrm {jets}$ contribution, given $Z \,\mathtt + \,\mathrm {jets}$ production in the same phase space, or vice versa. The $R_{\mathrm {jets}}$ measurement may also be sensitive to direct contributions from new particle production, if the new particles decay via $W$ or $Z$ bosons [1]. New physics phenomena are generally expected to appear in various topologies with high-momentum jets or high jet multiplicities, highlighting the importance of studying QCD effects in those regions of phase space.

The ATLAS collaboration performed the first measurement of $R_{\mathrm {jets}}$ as a function of the jet transverse momentum in events with exactly one jet in proton–proton collisions at $\sqrt{s}=7\,{\mathrm {\ TeV}}$, using a data sample corresponding to an integrated luminosity of $33\,\mathrm{pb}^{-1}$ [2]. This result demonstrated that the precision obtained in such a measurement is sufficient to be sensitive to the QCD effects mentioned above. The CMS collaboration performed an $R_{\mathrm {jets}}$ measurement of the jet multiplicity in vector-boson production with up to four associated jets, based on a similar dataset corresponding to an integrated luminosity of $36\,\mathrm{pb}^{-1}$ in $pp$ collisions collected at $\sqrt{s}=7\,{\mathrm {\ TeV}}$ [3]. The results reported in this paper  are based on a dataset corresponding to an integrated luminosity of $4.6\,\mathrm{fb}^{-1}$, collected with the ATLAS detector during the 2011 $pp$ collision run of the LHC at $\sqrt{s} = 7\,{\mathrm {\ TeV}}$. This dataset is over a hundred times larger than the one used in previously published results, allowing improved precision over a much larger region of phase space as well as the study of previously inaccessible differential distributions.

The $R_{\mathrm {jets}}$ measurement is done for the electron and muon decay channels of the $W$ and $Z$ bosons for jets with transverse momentum ${p}_\mathrm{T} > 30\,{\mathrm {\ GeV}}$ and rapidity $|y| < 4.4$.^2^ The measurements of the electron and muon channels are performed in slightly different phase spaces and combined in a common phase space defined in terms of the ${p}_\mathrm{T}$ and pseudorapidity $\eta$ of the leptons, the invariant mass of the $Z$ boson, the angular separation between the two leptons^3^ of the $Z$ boson decay, and the transverse mass^4^ of the $W$ boson, as presented in Table 1. The $W$ and $Z$ selections are based on the $W\,\mathtt + \,\mathrm {jets}$ and $Z \,\mathtt + \,\mathrm {jets}$ cross-section measurements detailed in Ref. [4, 5], with a minor update in the $Z$ selection to further reduce the uncertainty on the $R_{\mathrm {jets}}$ measurement. In the results reported here, $R_{\mathrm {jets}}$ is measured as a function of the inclusive and exclusive jet multiplicity (${N}_{\mathrm {jets}}$) up to four jets. An extensive set of differential measurements is also presented, in which $R_{\mathrm {jets}}$ is measured as a function of the transverse momentum and the rapidity of the leading jet, which is the one with largest transverse momentum, in events with at least one jet. The ratio $R_{\mathrm {jets}}$ is also presented as a function of the transverse momentum and rapidity of the second and third leading jets in events with at least two or three jets respectively. A set of differential measurements as a function of dijet observables in events with at least two jets is presented. The measurement of $R_{\mathrm {jets}}$ as a function of the summed scalar ${p}_\mathrm{T}$ of the jets (${S}_\mathrm{T}$) for different jet multiplicities is also reported. The results are compared to several Monte Carlo generators and with next-to-leading-order pQCD predictions corrected for non-perturbative effects.

**Table 1:** Particle-level phase space of the present $R_{\mathrm {jets}}$ measurement

Lepton ${p}_\mathrm{T}$ and pseudorapidity $\eta$	${p}_\mathrm{T} > 25\,{\mathrm {\ GeV}}$, $|\eta | < 2.5$
$W$ transverse mass and neutrino ${p}_\mathrm{T}$	${m}_{\mathrm {T}} > 40\,{\mathrm {\ GeV}}$, ${p}_\mathrm{T} > 25\,{\mathrm {\ GeV}}$
$Z$ invariant mass and lepton–lepton angular separation	$66 < {m}_{\ell \ell } < 116\,{\mathrm {\ GeV}}$, $\Delta R _{\ell \ell }>0.2$
Jet ${p}_\mathrm{T}$, rapidity and jet–lepton angular separation	${p}_\mathrm{T} > 30 {\mathrm {\ GeV}}$, $|y| < 4.4$, $\Delta R _{j\ell } > 0.5$

The paper  is organized as follows. The experimental setup is described in Sect. 2. Section 3 provides details on the simulations used in the measurement, and Sect. 4 discusses the event selection. The estimation of background contributions is described in Sect. 5, and the procedure used to correct the measurements for detector effects is described in Sect. 6. The treatment of the systematic uncertainties is described in Sect. 7. Section 8 discusses the combination of the electron and muon results. Section 9 provides details on the NLO pQCD predictions. Finally, Sect. 10 discusses the results, and Sect. 11 presents the conclusions.

## The ATLAS detector

The ATLAS detector [6] is a multi-purpose detector with a symmetric cylindrical geometry and nearly $4\pi$ coverage in solid angle. The collision point is surrounded by inner tracking devices followed by a superconducting solenoid providing a 2 T magnetic field, a calorimeter system, and a muon spectrometer. The inner tracker provides precision tracking of charged particles for pseudorapidities $|\eta | < 2.5$. It consists of silicon pixel and microstrip detectors and a straw-tube transition radiation tracker. The calorimeter system has liquid argon (LAr) or scintillator tiles as active media. In the pseudorapidity region $|\eta | < 3.2$, high-granularity LAr electromagnetic (EM) sampling calorimeters are used. An iron/scintillator tile calorimeter provides hadronic coverage for $|\eta | < 1.7$. The endcap and forward regions, spanning $1.5<|\eta | <4.9$, are instrumented with LAr calorimeters for both the EM and hadronic measurements. The muon spectrometer consists of three large superconducting toroids, each comprising eight coils, and a system of trigger chambers and precision tracking chambers that provide triggering and tracking capabilities in the ranges $|\eta | < 2.4$ and $|\eta | < 2.7$ respectively.

The ATLAS trigger system uses three consecutive levels. The Level-1 triggers are hardware-based and use coarse detector information to identify regions of interest, whereas the Level-2 triggers are based on fast online data reconstruction algorithms. Finally, the Event Filter triggers use offline data reconstruction algorithms.

## Monte Carlo simulation

Simulated event samples were used to correct the measured distributions for detector effects and acceptance, to determine some background contributions and to correct theory calculations for non-perturbative effects. Signal samples of $W (\rightarrow \ell \nu) \mathtt +$jets and $Z (\rightarrow \ell \ell) \mathtt +$jets (where $\ell = e, \mu)$) events were generated with ALPGEN v2.13 [7], with up to five additional partons in the final state. It was interfaced to HERWIG  v6.520 [8] for parton showering and fragmentation, with JIMMY  v4.31  [9] for contributions from multi-parton interactions and with PHOTOS [10] to calculate final-state QED radiation. The CTEQ6L1  [11] PDFs were used with the AUET2-CTEQ6L1 tune [12], a set of specific non-perturbative event generation parameter values. Similar samples were produced with ALPGEN  v2.14 interfaced to PYTHIA v6.425 [13] using the PERUGIA2011C  [14] tune and PHOTOS. They were used to estimate the uncertainties on non-perturbative corrections for parton-level NLO pQCD predictions. An additional set of signal samples was generated with SHERPA v1.4.1 [15, 16] and CT10 PDFs [17]. Top quark pair production ($t\bar{t}$) was simulated with ALPGEN and HERWIG+JIMMY, in the same configuration as for the signal samples. Additional $t\bar{t}$ samples were generated with the POWHEG-BOX generator v1.0 [18], using the CT10 next-to-leading order (NLO) PDFs and interfaced to PYTHIA  v6.425. These additional samples were reserved for the evaluation of the systematic uncertainties. Single top-quark production, including $Wt$ production, was modelled with AcerMC 3.8 [19] interfaced to PYTHIA and MRST LO* PDFs [20]. The diboson production processes $W^+W^-, WZ$, and $ZZ$ were generated with HERWIG  v6.510 and JIMMY  v4.3 using the MRST LO* PDFs [20] and the AUET2-LO* tune [12].

The generated Monte Carlo (MC) samples were overlaid with additional inelastic $pp$ scattering events generated with PYTHIA v6.425, following the distribution of the average number of $pp$ interactions in the selected data. The samples were then passed through the simulation of the ATLAS detector based on GEANT4  [21, 22] and through the related trigger simulation.

All samples were normalized to the inclusive cross section calculated at the highest pQCD order available. The $W/Z\mathtt +$jets signal samples were normalized to the next-to-next-to-leading-order (NNLO) pQCD inclusive Drell–Yan predictions calculated with the FEWZ [23] program and the MSTW2008 NNLO PDFs [24]. The $t\bar{t}$ samples were normalized to the cross section calculated at NNLO+NNLL in Refs. [25–30], and the diboson samples were normalized to cross sections calculated at NLO using MCFM [31] with the MSTW2008 PDF set.

The simulated events were reconstructed and analysed with the same analysis chain as the data. Scale factors were applied to the simulated samples to correct the lepton trigger, reconstruction, and identification efficiencies to match those measured in data.

## Event selection

**Table 2:** Kinematic event selection criteria for $W(\rightarrow \ell \nu )\,\mathtt + \,\mathrm {jets}$ and $Z \,(\rightarrow \ell \ell ) \,\mathtt + \,\mathrm {jets}$ event samples

	Electron selection	Muon selection
Lepton ${p}_\mathrm{T}$	${p}_\mathrm{T} > 25$ GeV	${p}_\mathrm{T} > 25$ GeV
Lepton pseudorapidity	$|\eta |<2.47$ (excluding $1.37<|\eta |<1.52$)	$|\eta |<2.4$
$W\rightarrow \ell \nu$ event selection		
$Z$ veto	Exactly one selected lepton	
Missing transverse momentum	$E_{\mathrm {T}}^{\mathrm {miss}} > 25$ GeV	
Transverse mass	${m}_{\mathrm {T}} > 40$ GeV	
$Z\rightarrow \ell \ell$ event selection		
Multiplicity	Exactly two selected leptons	
Charge	Opposite sign	
Invariant mass	$66 < m_{\ell \ell } < 116$ GeV	
Separation	$\Delta R_{\ell \ell }>0.2$	
Jet selection		
Transverse momentum	${p}_\mathrm{T} > 30$ GeV	
Jet rapidity	|$y| < 4.4$	
Jet–lepton angular separation	$\Delta R _{\ell \mathrm{j}} > 0.5$	

The data samples considered in this paper  correspond to a total integrated luminosity of $4.6\,\mathrm{fb}^{-1}$, with an uncertainty of 1.8 $\%$ [32]. Table 2 summarizes the kinematic requirements for leptons, $W$ bosons, $Z$ bosons, and jets. The selection criteria for $W$ boson candidates were defined using the largest possible coverage of the ATLAS detector for electrons, muons and jets. The selection criteria for $Z$ boson candidates were modified with respect to those in Ref. [5], to be as similar as possible to the $W$ boson selection in order to maximize the cancellation of uncertainties in the $R_{\mathrm {jets}}$ measurement: triggers requiring at least one lepton were employed, the minimum lepton transverse momentum was raised from 20 GeV to 25 GeV , tighter criteria were used to identify electrons and slightly looser requirements were placed on the second leading lepton with respect to the leading one.

The data were collected using single-electron or single-muon triggers, employing the same requirements for the $W$ and $Z$ data selections. Electron-channel events were selected using a trigger that required the presence of at least one electron candidate, formed by an energy cluster consistent with an electromagnetic shower in the calorimeter and associated to an inner detector track. Electron candidates were required to have a reconstructed transverse energy above $20\,{\mathrm {\ GeV}}$ or $22\,{\mathrm {\ GeV}}$, depending on the trigger configuration of the different data periods. Muon-channel events were recorded using a trigger that required the presence of at least one muon candidate with transverse momentum above $18\,{\mathrm {\ GeV}}$. Lepton trigger thresholds were low enough to ensure that leptons with ${p}_\mathrm{T} > 25\,{\mathrm {\ GeV}}$ lie on the trigger efficiency plateau.

Events were required to have a primary vertex, defined as the vertex in the event with the highest summed ${p}_\mathrm{T} ^2$ of all associated tracks, among vertices with at least three tracks.

Electrons were reconstructed by matching clusters of energy found in the electromagnetic calorimeter to tracks reconstructed in the inner detector. Candidate electrons had to satisfy the “tight” quality requirements defined in Ref. [33], which include requirements on the calorimeter shower shape, track quality, and association of the track with the energy cluster found in the calorimeter. Electron candidates had to have ${p}_\mathrm{T} > 25\,{\mathrm {\ GeV}}$ and $|\eta |<2.47$, where the transition region between barrel and endcap electromagnetic calorimeter sections at $1.37<|\eta |<1.52$ was excluded.

Muons were reconstructed from track segments in the muon spectrometer that were matched with tracks in the inner detector [34], and were required to have ${p}_\mathrm{T} > 25$ GeV and $|\eta |<2.4$. To suppress particles from hadron decays, the leading muon had to be consistent with originating from the primary vertex by requiring $|d_0/\sigma (d_0)| < 3.0$, where $d_0$ is the transverse impact parameter of the muon and $\sigma (d_0)$ is its uncertainty.

In order to suppress background from multi-jet events where a jet is misidentified as a lepton, the leading lepton was required to be isolated. An additional ${p}_\mathrm{T}$- and $\eta$-dependent requirement on a combination of calorimeter and track isolation variables was applied to the leading electron, in order to yield a constant efficiency across different momentum ranges and detector regions, as detailed in Ref. [35]. The track-based isolation uses a cone size of $\Delta R = 0.4$ and the calorimeter-based isolation uses a cone size of $\Delta R = 0.2$. The actual isolation requirements range between 2.5 GeV and 4.5 GeV for the calorimeter-based isolation and between 2.0 GeV and 3.0 GeV for the track-based isolation. For muon candidates, the scalar sum of the transverse momenta of tracks within a cone of size $\Delta R = 0.2$ around the leading muon had to be less than $10\,\%$ of its transverse momentum.

Reconstructed $W$ candidates were required to have exactly one selected lepton. The missing transverse momentum in the event had to have a magnitude $E_{\mathrm {T}}^{\mathrm {miss}}$ greater than $25$ GeV , and the transverse mass $m_T$ had to be greater than $40$ GeV . The magnitude and azimuthal direction of the missing transverse momentum are measured from the vector sum of the transverse momenta of calibrated physics objects and additional soft calorimeter deposits [36]. Reconstructed $Z$ candidates were required to have exactly two selected leptons of the same flavour with opposite charge. Their invariant mass $m_{\ell \ell }$ had to be in the range $66 \le m_{\ell \ell } \le 116\,{\mathrm {\ GeV}}$ and the leptons had to be separated by $\Delta R_{\ell \ell } > 0.2$.

Jets were reconstructed using the anti-$k_{t}$ algorithm [37] with a distance parameter $R=0.4$ on topological clusters of energy in the calorimeters [38]. Jets were required to have a transverse momentum above $30\, {\mathrm {\ GeV}}$ and a rapidity of $|y| < 4.4$. Jets within $\Delta R =0.5$ of a selected lepton were removed. The energy and the direction of reconstructed jets were corrected to account for the point of origin, assumed to be the primary vertex, and for the bias introduced by the presence of additional $pp$ interactions in the same bunch crossing (“pile-up”). The jet energy was then calibrated to account for the different response of the calorimeters to electrons and hadrons and for energy losses in un-instrumented regions by applying correction factors derived from simulations. A final calibration, derived from in-situ techniques using Z+jet balance, $\gamma$+jet balance and multi-jet balance, was applied to the data to reduce residual differences between data and simulations [39].

In order to reject jets from pile-up, a jet selection was applied based on the ratio of the summed scalar ${p}_\mathrm{T}$ of tracks originating from the primary vertex and associated with the jet to the summed ${p}_\mathrm{T}$ of all tracks associated with the jet. Jets were selected if this ratio was above $0.75$. This criterion was applied to jets within $|\eta |<2.4$, so that they are inside the inner tracker acceptance. Comparison between data and simulation for various data periods confirmed that the residual impact of pile-up on the distribution of the jet observables in this analysis is well modelled by the simulation.

The numbers of $W\,\mathtt + \,\mathrm {jets}$ and $Z \,\mathtt + \,\mathrm {jets}$ candidate events in the electron and muon channels for each jet multiplicity are shown in Tables 3 and 4, together with the corresponding numbers of predicted events. The expected fraction of predicted events from signal and each background source, determined as described in the next section, is also shown.

## Background estimation

Background processes to $W$ and $Z$ boson production associated with jets can be classified into three categories. The first category, referred to as electroweak background, consists of diboson production, vector-boson production with subsequent decay to $\tau$-leptons, and “cross-talk” background, in which the signal $W\,\mathtt + \,\mathrm {jets}$ ($Z \,\mathtt + \,\mathrm {jets}$) production appears as background in the $Z \,\mathtt + \,\mathrm {jets}$ ($W\,\mathtt + \,\mathrm {jets}$) sample. These background contributions are relatively small (about 10 % in the $W\,\mathtt + \,\mathrm {jets}$ electron channel, about 6 % in the $W\,\mathtt + \,\mathrm {jets}$ muon channel, and about 1 % in $Z \,\mathtt + \,\mathrm {jets}$, as shown in Tables 3 and 4) and were thus estimated using simulated event samples.

The second category consists of events where the leptons are produced in decays of top quarks. The $t\bar{t}$ component completely dominates the background contribution to $W\,\mathtt + \,\mathrm {jets}$ events at high jet multiplicities, amounting to approximately $20\,\%$ of the sample with $W\mathtt + \ge 3 {\mathrm{jets}}$ and increasing to approximately $45\,\%$ for events with four selected jets. The effect is less dramatic in $Z \,\mathtt + \,\mathrm {jets}$ events, where the $t\bar{t}$ background contributes about $5\,\%$ to the sample of events with ${Z\mathtt + \ge 3\, \mathrm{jets}}$ and about $10\,\%$ to the sample with four jets. The background contribution from single top-quark production is about $4\,\%$ of the sample in $W\,\mathtt + \,\mathrm {jets}$ events for events with three or four jets, and smaller at lower jet multiplicities. This contribution is even smaller in $Z \,\mathtt + \,\mathrm {jets}$ events. Contributions from $t\bar{t}$ events to $W\,\mathtt + \,\mathrm {jets}$ candidates with at least three jets, where this background dominates, were estimated with a data-driven method as described below in order to reduce the overall uncertainty. The $t\bar{t}$ contributions to $W\,\mathtt + \,\mathrm {jets}$ candidates with fewer than three jets and to $Z \,\mathtt + \,\mathrm {jets}$ events were estimated using simulated event samples, as are the contributions from single top quarks.

The third category of background, referred to as multi-jet background, comes from events in which hadrons mimic the signature of an isolated lepton. In the electron channel this includes photon conversion processes, typically from the decay of neutral pions, narrow hadronic jets and real electrons from the decay of heavy-flavour hadrons. In the muon channel, the multi-jet background is primarily composed of heavy-flavour hadron decay processes. This background category dominates at low jet multiplicity in $W\,\mathtt + \,\mathrm {jets}$ events, amounting to $11\, \%$ of the selected sample in both the electron and muon channels for events with one jet. Data-driven techniques were used to estimate this background contribution to both the $W\,\mathtt + \,\mathrm {jets}$ and $Z \,\mathtt + \,\mathrm {jets}$ candidate events, as described below. The methods employed to estimate background contributions with data-driven techniques in this analysis are very similar between candidate events with $W$ bosons and $Z$ bosons and between electron and muon channels.

### $t\bar{t}$ background

The $t\bar{t}$ background is the dominant background contribution to $W\,\mathtt + \,\mathrm {jets}$ events with at least three jets, since each top quark predominantly decays as $t \rightarrow Wb$. The size of the $t\bar{t}$ contribution was estimated with a maximum-likelihood fit to the data.

The $t\bar{t}$ template in this fit was derived from a top–quark-enhanced data sample by requiring, in addition to the selection criteria given in Table 2, at least one $b$-tagged jet in the event, as determined by the MV1 $b$-tagging algorithm of Ref. [40]. The chosen MV1 algorithm working point has a $b$-tagging efficiency of 70 $\%$. This data sample is contaminated with $W$ signal events and electroweak and multi-jet backgrounds, amounting to about 40 $\%$ in events with three jets and 25 $\%$ in events with four jets. The contribution from $W$ signal events and electroweak background was estimated using simulation. The multi-jet contribution to the top-enriched sample was estimated using the multi-jet background estimation method as outlined in the last part of this section, but with an additional $b$-tagging requirement. Potential biases in the $t\bar{t}$ templates extracted from data were investigated using simulated $t\bar{t}$ events. Since $b$-tagging is only available for jets within $|\eta | < 2.4$ where information from the tracking detectors exists, the $b$-tagging selection biases some of the kinematic distributions, most notably the jet rapidity distribution. To account for this, ALPGEN $t\bar{t}$ simulations were used to correct for any residual bias in the differential distributions; the maximum correction is 30 %.

The number of $t\bar{t}$ events was extracted by fitting a discriminant distribution to the sum of three templates: the top-enriched template after subtracting the contaminations discussed above, the multi-jet template (determined as described below) and the template obtained from simulation of the $W\,\mathtt + \,\mathrm {jets}$ signal and the other background sources. The chosen discriminant was the transformed aplanarity, given by $\exp (-8A)$, where ${A}$ is the aplanarity defined as 1.5 times the smallest eigenvalue of the normalized momentum tensor of the leptons and all the jets passing the selection [41]. This discriminant provides the best separation between $t\bar{t}$ and the $W\,\mathtt + \,\mathrm {jets}$ signal. The fit to the transformed aplanarity distribution was done in the range $0.0$–$0.85$ in each exclusive jet multiplicity of three or more.

Since the top-enriched sample is a sub-sample of the signal sample, statistical correlation between the two samples is expected. Its size was estimated using pseudo-datasets by performing Poisson variations of the signal and top-enriched samples. To account for this correlation, the uncertainty on the fit was increased by 15 $\%$ for events with three jets and about 30 $\%$ for events with four jets.

###  Multi-jet background

The multi-jet background contribution to the $W\,\mathtt + \,\mathrm {jets}$ selected events was estimated with a template fit method using a sample enriched in multi-jet events. The templates of the multi-jet background for the fit were extracted from data, by modifying the lepton isolation requirements in both the electron and muon channels, in order to select non-isolated leptons. The templates of the signal, the $t\bar{t}$ background, and the electroweak background were obtained from simulation. These templates were then normalized by a fit to the $E_{\mathrm {T}}^{\mathrm {miss}}$ distribution after all signal requirements other than the requirement on $E_{\mathrm {T}}^{\mathrm {miss}}$ were applied.

To select an electron-channel data sample enriched in multi-jet events, dedicated electron triggers based on loose requirements were used (as defined in Ref. [33]), along with additional triggers based on loose electron and jet selection criteria. The background template distributions were built from events for which the identification requirements of the nominal electron selection failed, in order to suppress signal contamination in the template. Candidate electrons were also required to be non-isolated in the calorimeter, i.e. were required to have an energy deposition in the calorimeter in a cone of size $\Delta R < 0.3$ centred on their direction greater than 20 % of their total transverse energy. This selection results in a data sample highly enriched in jets misidentified as electrons. As the luminosity increased during the course of 2011, the trigger selections were adjusted to cope with the increasing trigger rates. In order to build multi-jet template distributions that provide a good representation of the pile-up conditions of the selected data sample, these template distributions were extracted from two distinct data periods with high and low pile-up conditions. The background templates extracted from the two different data periods were fitted separately and then combined into an overall multi-jet estimate.

To select the multi-jet sample in the muon channel, muon candidates were required to be non-isolated. The sum of transverse momenta of tracks in a cone of size $\Delta R < 0.2$ centred on the muon-candidate direction had to be between $10\,\%$ and $50\,\%$ of the muon transverse momentum. The contamination from $W$ signal events and electroweak and top backgrounds to the multi-jet sample was subtracted using simulation. It amounts to $1.4\, \%$ for events with one jet and $4.8\, \%$ for events with four jets.

The number of multi-jet background events was obtained for each jet multiplicity in the electron and muon channels by fitting the $E_{\mathrm {T}}^{\mathrm {miss}}$ distribution obtained from the $W\,\mathtt + \,\mathrm {jets}$ data candidate events (selected before the application of the $E_{\mathrm {T}}^{\mathrm {miss}}$ requirement) to the multi-jet template and a template of signal and electroweak and $t\bar{t}$ backgrounds derived from simulations. The fit range was chosen to ensure significant contributions from both templates, in order to guarantee fit stability under systematic variations described in Sect. 7. The $E_{\mathrm {T}}^{\mathrm {miss}}$ distribution was fitted in the range $15\, {\mathrm {\ GeV}}$ to $80\,{\mathrm {\ GeV}}$ in the electron channel and in the range $15\, {\mathrm {\ GeV}}$ to $70\,{\mathrm {\ GeV}}$ in the muon channel.

The multi-jet background contribution to the $Z \,\mathtt + \,\mathrm {jets}$ selected candidates was estimated using a template fit method similar to the procedure used in the $W\,\mathtt + \,\mathrm {jets}$ case. In the electron channel, the template distributions for the multi-jet background were constructed from a data sample collected with electron triggers looser than those used for the nominal $Z\rightarrow e e$ selection. Electrons were then required to satisfy the loose offline identification criteria (as defined in Ref. [33]) but fail to meet the nominal criteria. In the muon channel, the multi-jet template distributions for the multi-jet background were obtained from the nominal signal data sample, after relaxing the impact parameter significance requirement applied to $Z\rightarrow \mu \mu$ events candidates, and selecting events that did not satisfy the isolation criteria applied in the signal selection. The number of multi-jet background events was obtained for each exclusive jet multiplicity by fitting the dilepton invariant mass distribution $m_{\ell \ell }$ in an extended range, $50 < m_{\ell \ell } < 140$ GeV , excluding the $Z$-peak region itself, after all other signal requirements were applied. Due to statistical limitations for jet multiplicities greater than two jets, the normalisation factor obtained from the two-jet bin was consistently applied to the templates for higher jet multiplicities. Potential bias in this procedure was accounted for in the systematic uncertainty estimate.

The evaluation of the systematic uncertainties for each background source is explained in Sect. 7.

**Table 3:** The contribution of signal and background from various sources, expressed as a fraction of the total number of expected events for the $W(\rightarrow e \nu )\,\mathtt + \,\mathrm {jets}$ and $Z \,(\rightarrow e e) \,\mathtt + \,\mathrm {jets}$ selection as a function of jet multiplicity ${N}_{\mathrm {jets}}$ together with the total numbers of expected and observed events

$N_{\text{ jets }}$	0	1	2	3	4
Fraction [%]	$W (\rightarrow e\nu )+$ jets				
$W\rightarrow e\nu$	94	78	73	58	37
$Z\rightarrow ee$	0.30	7.5	6.6	6.8	5.4
$$t\bar{t}$$	$$<0.1 $$	0.30	3.4	18	46
Multi-jet	4	11	12	11	6.9
Electroweak (without $Z\rightarrow ee$)	1.9	2.6	3.3	3	1.9
Single top	$$<0.1 $$	0.30	1.7	3.5	3.9
Total predicted	11 100 000 $\pm$ 640 000	1 510 000 $\pm$ 99 000	354 000 $\pm$ 23 000	89 500 $\pm$ 5600	28 200 $\pm$ 1400
Data observed	10 878 398	1 548 000	361 957	91 212	28 076
Fraction [%]	$Z (\rightarrow ee)$ + jets				
$Z\rightarrow ee$	100	99	96	93	90
$W\rightarrow e\nu$	$$<0.1\;\ $$	$$<0.1\;\ $$	$$<0.1\;\ $$	$$<0.1\;\ $$	$$<0.1\;\ $$
$t\bar{t}$	$$<0.1\;\ $$	0.20	1.9	4.6	7.8
Multi-jet	0.20	0.20	0.40	0.50	0.50
Electroweak (without $W\rightarrow e\nu$)	0.10	0.50	1.3	1.4	1.2
Single top	$$<0.1 $$	$$<0.1 $$	0.10	0.20	0.10
Total predicted	754 000 $\pm$ 47 000	96 500 $\pm$ 6900	22 100 $\pm$ 1700	4700 $\pm$ 930	1010 $\pm$ 93
Data observed	761 280	99 991	22 471	4 729	1050

**Table 4:** The contribution of signal and background from various sources, expressed as a fraction of the total number of expected events for the $W(\rightarrow \mu \nu )\,\mathtt + \,\mathrm {jets}$ and $Z \,(\rightarrow \mu \mu ) \,\mathtt + \,\mathrm {jets}$ selection as a function of jet multiplicity ${N}_{\mathrm {jets}}$ together with the total numbers of expected and observed events

$N_{\text{ jets }}$	0	1	2	3	4
Fraction [%]	$W (\rightarrow \mu \nu )$ + jets				
$W\rightarrow \mu \nu$	93	82	78	62	40
$Z\rightarrow \mu \mu$	3.4	3.5	3.5	3	2
$t\bar{t}$	$$<0.1\;\ $$	0.20	3.1	19	46
Multi-jet	1.5	11	10	9.5	6.8
Electroweak (without $Z\rightarrow \mu \mu$)	1.9	2.7	3.4	2.9	1.9
Single top	$$<0.1\;\ $$	0.20	1.7	3.4	3.8
Total predicted	13 300 000 $\pm$ 770 000	1 710 000 $\pm$ 100 000	384 000 $\pm$ 24 000	96 700 $\pm$ 6100	30 100 $\pm$ 1600
Data observed	13 414 400	1 758 239	403 146	99 749	30 400
Fraction [%]	$Z (\rightarrow \mu \mu )+$ jets				
$Z\rightarrow \mu \mu$	100	99	96	91	84
$W\rightarrow \mu \nu$	$$<0.1\;\ $$	0.10	0.10	0.20	0.20
$t\bar{t}$	$$<0.1\;\ $$	0.30	2.2	6.1	13
Multi-jet	0.30	0.50	0.90	1.1	1.7
Electroweak (without $W\rightarrow \mu \nu$)	0.10	0.50	1.3	1.4	1.1
Single top	$$<0.1\;\ $$	$$<0.1\;\ $$	0.10	0.20	0.20
Total predicted	1 300 000 $\pm$ 79 000	168 000 $\pm$ 12 000	37 800 $\pm$ 2800	8100 $\pm$ 660	1750 $\pm$ 160
Data observed	1 302 010	171 200	38 618	8397	1864

## Corrections for detector effects

The signal event yields were determined by subtracting the estimated background contributions from the data. After background subtraction, the resulting distributions were corrected for detector effects such that distributions at particle level were obtained. The correction procedure based on simulated samples corrects for jet, $W$ and $Z$ selection efficiency, resolution effects and residual mis-calibrations. While $W\,\mathtt + \,\mathrm {jets}$ and $Z \,\mathtt + \,\mathrm {jets}$ events were separately corrected before forming $R_{\mathrm {jets}}$, the systematic uncertainties were estimated for the ratio itself, as explained in the next section.

At particle level, the lepton kinematic variables in the MC-generated samples were computed using final-state leptons from the $W$ or $Z$ boson decay. Photons radiated by the boson decay products within a cone of size $\Delta R$ $= 0.1$ around the direction of a final-state lepton were added to the lepton, and the sum is referred to as the “dressed” lepton. Particle-level jets were identified by applying the anti-$k_{t}$ algorithm with $R = 0.4$ to all final-state particles with a lifetime longer than $30\,\mathrm {ps}$, whether produced directly in the proton–proton collision or from the decay of particles with shorter lifetimes. Neutrinos, electrons, and muons from decays of the $W$ and $Z$ bosons, as well as collinear photons included in the “lepton dressing procedure” were excluded by the jet reconstruction algorithm. The phase-space requirements match the selection criteria defining the data candidate events, as presented in Table 2, in order to limit the dependence of the measurement results on theoretical assumptions. 

The correction was implemented using an iterative Bayesian method of unfolding [42]. Simulated events are used to generate for each distribution a response matrix to account for bin-to-bin migration effects between the reconstruction-level and particle-level distributions. The Monte Carlo particle-level prediction is used as initial prior to determine a first estimate of the unfolded data distribution. For each further iteration, the previous estimate of the unfolded distribution is used as a new input prior. Bin sizes in each distribution were chosen to be a few times larger than the resolution of the corresponding variable. The ALPGEN $W\,\mathtt + \,\mathrm {jets}$ and $Z \,\mathtt + \,\mathrm {jets}$ samples provide a satisfactory description of distributions in data and were employed to perform the correction procedure. The number of iterations was optimized to find a balance between too many iterations, causing high statistical uncertainties associated with the unfolded spectra, and too few iterations, which increase the dependency on the Monte Carlo prior. The optimal number of iterations is typically between one and three, depending on the observable. Since the differences in the unfolded results are negligible over this range of iterations, two iterations were used consistently for unfolding each observable.

## Systematic uncertainties

**Table 5:** Systematic uncertainties in percent on the measured $W\,\mathtt + \,\mathrm {jets}$ / $Z \,\mathtt + \,\mathrm {jets}$ cross-section ratio in the electron and muon channels as a function of the inclusive jet multiplicity ${N}_{\mathrm {jets}}$

$N_\mathrm {jets}$	$\ge 0$	$\ge 1$	$\ge 2$	$\ge 3$	$\ge 4$
$$ (W \rightarrow e\nu ) / (Z \rightarrow ee)$$					
Electron	0.89	0.92	0.93	0.97	1.0
JES	0.094	2.0	2.0	3.5	5.7
JER	0.25	2.4	3.5	4.3	6.4
$E_{\mathrm {T}}^{\mathrm {miss}}$	0.19	1.7	1.2	1.2	1.0
$t\bar{t}$	0.024	0.23	1.0	4.9	14
Multi-jet	0.81	1.6	1.5	2.2	6.2
Other backgrounds	0.12	0.57	0.58	0.76	1.0
Unfolding	0.20	0.56	0.86	1.2	1.4
Luminosity	0.062	0.26	0.27	0.34	0.44
Total	1.3	4.1	4.8	8.2	18
$$ (W \rightarrow \mu \nu ) / (Z \rightarrow \mu \mu )$$					
Muon	1.1	1.2	1.1	0.86	0.87
JES	0.10	0.84	0.71	1.8	2.6
JER	0.094	1.6	1.8	2.6	4.2
$E_{\mathrm {T}}^{\mathrm {miss}}$	0.30	1.0	0.94	0.97	0.99
$t\bar{t}$	0.018	0.18	0.87	4.3	12
Multi-jet	0.20	0.60	1.1	1.7	2.7
Other backgrounds	0.21	0.24	0.28	0.42	0.60
Unfolding	0.22	0.59	0.90	1.2	1.2
Luminosity	0.10	0.12	0.11	0.088	0.023
Total	1.2	2.5	3.0	5.9	13

One of the advantages of measuring $R_{\mathrm {jets}}$ is that systematic uncertainties that are positively correlated between the numerator and denominator cancel at the level of their correlations (higher correlations result in larger cancellations). The impact on the ratio of a given source of uncertainty was estimated by simultaneously applying the systematic variation due to this source to both the $W\,\mathtt + \,\mathrm {jets}$ and $Z \,\mathtt + \,\mathrm {jets}$ events and repeating the full measurement chain with the systematic variations applied. This included re-estimating the data-driven background distributions after the variations had been applied.

Since the uncertainties were found to be symmetric within the statistical fluctuations, the resulting systematic uncertainties on the $R_{\mathrm {jets}}$ measurements were fully symmetrized by taking the average value of the upwards and downwards variations.

Uncertainty sources affecting the $R_{\mathrm {jets}}$ measurements can be assigned to one of the following categories: jet measurements, lepton measurements, missing transverse momentum measurement, unfolding procedure, data-driven background estimates and simulation-based background estimates. These sources of uncertainty feature significant correlations between $W\,\mathtt + \,\mathrm {jets}$ and $Z \,\mathtt + \,\mathrm {jets}$ processes, which have been fully accounted for as explained above. The systematic uncertainties on the $t\bar{t}$ and multi-jet background estimates were considered to be uncorrelated between the $W\,\mathtt + \,\mathrm {jets}$ and $Z \,\mathtt + \,\mathrm {jets}$ selections. The uncertainty on the integrated luminosity was propagated through all of the background calculations and treated as correlated between $W\,\mathtt + \,\mathrm {jets}$ and $Z \,\mathtt + \,\mathrm {jets}$ so that it largely cancels in the ratio. The contributions from each of the sources mentioned above and the total systematic uncertainties were obtained by adding in quadrature the different components, and are summarized in Table 5. The total uncertainty on $R_{\mathrm {jets}}$ as a function of the inclusive jet multiplicity ranges from $4\,\%$ for ${N}_{\mathrm {jets}} \ge 1$ to 18$\,\%$ for ${N}_{\mathrm {jets}} \ge 4$ in the electron channel and from 3 % for ${N}_{\mathrm {jets}} \ge 1$ to 13 % for ${N}_{\mathrm {jets}} \ge 4$ in the muon channel.

Jet-related systematic uncertainties are dominated by the uncertainty on the jet energy scale (JES) and resolution (JER). The JES uncertainty was derived via in-situ calibration techniques, such as the transverse momentum balance in $Z \,\mathtt + \,\mathrm {jets}$, multi-jet and $\gamma -$jet events, for which a comparison between data and simulation was performed [39]. The JER uncertainty was derived from a comparison of the resolution measured in dijet data events using the bisector method [38], and the same approach was applied to simulated dijet events. The JER and JES uncertainties are highly correlated between $W\,\mathtt + \,\mathrm {jets}$ and $Z \,\mathtt + \,\mathrm {jets}$ observables and are thus largely suppressed compared to the individual measurements. They are nevertheless the dominant systematic uncertainties in the cases where there are one or two jets in the events. The cancellation is not perfect because any changes in JES and JER are consistently propagated to the $E_{\mathrm {T}}^{\mathrm {miss}}$ measurement event-by-event. This causes larger associated migrations for the $W$ selection than for the $Z$ selection. In addition, the level of background in the $W\,\mathtt + \,\mathrm {jets}$ sample is larger, resulting in a larger jet uncertainty compared to the $Z \,\mathtt + \,\mathrm {jets}$ selection. The sum of JER and JES uncertainties on the $R_{\mathrm {jets}}$ measurement ranges from 3 % to 8 % in the electron channel and from 2 % to 5 % in the muon channel as ${N}_{\mathrm {jets}}$ ranges from 1 to 4. The difference between the two channels is due to the fact that the $Z\rightarrow ee$ background in the $W \rightarrow e\nu$ data sample is much larger than the corresponding $Z\rightarrow \mu \mu$ background in the $W \rightarrow \mu \nu$ sample, being about 7 % for candidate events with one jet in the electron channel compared to 3 % in the muon channel. The $Z\rightarrow ee$ background contaminates the $W \rightarrow e\nu$ sample because one electron can be misidentified as a jet, contributing to the JES and JER uncertainties. This contribution to the uncertainties does not cancel in $R_{\mathrm {jets}}$.

The uncertainty on the electron and muon selections includes uncertainties on the electron energy or muon momentum scale and resolution, as well as uncertainties on the scale factors applied to the simulations in order to match the electron or muon trigger, reconstruction and identification efficiencies to those in data. Any changes in lepton energy scale and resolution were consistently propagated to the $E_{\mathrm {T}}^{\mathrm {miss}}$ measurement. The energy- or momentum-scale corrections of the leptons were obtained from comparison of the $Z$-boson invariant mass distribution between data and simulations. The uncertainties on the scale factors have been derived from a comparison of tag-and-probe results in data and simulations [33, 34]. Each of these sources of uncertainty is relatively small in the $R_{\mathrm {jets}}$ measurement (about $1\%$ for $N_{\mathrm {jets}}$ ranging from 1 to 4 in both channels).

The uncertainties in $E_{\mathrm {T}}^{\mathrm {miss}}$ due to uncertainties in JES, JER, lepton energy scale and resolution were included in the values quoted above. A residual $E_{\mathrm {T}}^{\mathrm {miss}}$ uncertainty accounts for uncertainties on the energy measurement of clusters in the calorimeters that are not associated with electrons or jets. It was determined via in-situ measurements and comparisons between data and simulation [43]. These systematic uncertainties affect only the numerator of the ratio because no $E_{\mathrm {T}}^{\mathrm {miss}}$ cut was applied to $Z \mathtt +$ jets candidate events. The resulting uncertainty on the $R_{\mathrm {jets}}$ measurement is about $1\,\%$ for $N_{\mathrm {jets}}$ ranging from 1 to 4 in both channels.

The uncertainty on the unfolding has a component of statistical origin that comes from the limited number of events in each bin of the Monte Carlo inputs. This component was estimated from the root mean square of $R_{\mathrm {jets}}$ measurements obtained in a large set of pseudo-data generated independently from the $W\,\mathtt + \,\mathrm {jets}$ and $Z \,\mathtt + \,\mathrm {jets}$ Monte Carlo samples used to unfold the data. The Monte Carlo modelling uncertainty in the unfolding procedure was estimated using an alternative set of ALPGEN samples for which the nominal $W\,\mathtt + \,\mathrm {jets}$ and $Z \,\mathtt + \,\mathrm {jets}$ production was modelled by different theoretical parameter values. The MLM matching procedure [44], employed to remove the double counting of partons generated by the matrix element calculation and partons produced in the parton shower, uses a matching cone of size $R=0.4$ for matrix element partons of ${p}_\mathrm{T} > 20$ GeV . To determine how the choice of this cone size and the matching ${p}_\mathrm{T}$ scale impact the unfolded results, samples with variations of these parameters were used in the unfolding procedure. In addition, to account for the impact of changing the amount of radiation emitted from hard partons, ALPGEN Monte Carlo samples were generated with the renormalisation and factorisation scales set to half or twice their nominal value of $\sqrt{ m_{V}^2\mathtt + {{p}_\mathrm{T}}_{V}^2}$, where $V$ is the $W$ or $Z$ boson depending on the sample. The systematic uncertainty is the sum in quadrature of the differences with respect to the $R_{\mathrm {jets}}$ measurement obtained from the nominal samples. The overall uncertainty on the unfolding procedure ranges between $0.6\, \%$ and $1.4\, \%$ for $N_{\mathrm {jets}}$ ranging from 1 to 4.

For backgrounds estimated using simulation, the uncertainty on the cross-section calculation was taken into account. The combined impact of these uncertainties on the $R_{\mathrm {jets}}$ measurement is typically less than 1 $\%$ for the different jet multiplicities.

For $t\bar{t}$ predictions taken from the ALPGEN sample, the uncertainty on the cross-section calculation is considered, as well as a shape uncertainty by comparing to the POWHEG-BOX $t\bar{t}$ sample. The largest contribution to the total uncertainty from the data-driven $t\bar{t}$ estimate is from the statistical uncertainty on the fit. The systematic uncertainty on the data-driven $t\bar{t}$ estimate also covers uncertainties on the contamination of the background template by signal events, on the choice of fit range and other small uncertainties. The latter include the uncertainties on the $b$-tagging efficiencies and uncertainties on the bias in the $t\bar{t}$ distributions when applying the $b$-tagging. The uncertainty on the contribution from $W\mathtt +$ heavy-flavour events to the $t\bar{t}$ template, modelled by ALPGEN Monte Carlo samples, was evaluated by varying the $W\mathtt + c$ cross section and the combined $W\mathtt + cc$ and $W\mathtt + bb$ cross sections. The size of the variations is a factor of 0.9 and 1.3 respectively. These factors were obtained from fits to the data in two control regions, defined as one or two jets and at least one $b$-tagged jet. This uncertainty, which is $3\, \%$ of the number of $t\bar{t}$ events for $N_{\mathrm {jets}}\ge 3$, is largest at lower jet multiplicities where the contribution from $W\mathtt +$heavy flavour is most significant. The upper limit of the fit range in transformed aplanarity was varied from the nominal values of $0.85$ to $0.83$ or $0.87$. The $t\bar{t}$ uncertainty dominates for final states with high jet multiplicity due to its increasing contribution, which does not cancel in $R_{\mathrm {jets}}$. It amounts to an uncertainty of $14\, \%$ on the $R_{\mathrm {jets}}$ measurement in the electron channel and to an uncertainty of $12\, \%$ in the muon channel for events with at least four jets.

In the evaluation of the multi-jet background systematic uncertainties, various sources were taken into account. For the $W\mathtt +$jets selection, the uncertainty on the shape of the template distributions of the multi-jet background was studied by varying the lepton isolation requirement and identification definition. The nominal template fit range for $E_{\mathrm {T}}^{\mathrm {miss}}$ was also varied, within $10$ GeV up and down from the nominal limits. The distributions of the signal template were alternatively modelled by SHERPA instead of ALPGEN and the difference was taken as an uncertainty. The statistical uncertainty on the template normalisation factor from the fit was also included. Finally, to evaluate the uncertainty on the estimate of the multi-jet background to the $Z \,\mathtt + \,\mathrm {jets}$ events, the fit ranges and the modelling of the signal and of the electroweak contamination were varied in the same way as for the $W\,\mathtt + \,\mathrm {jets}$ events. The combined impact of these uncertainties on the $R_{\mathrm {jets}}$ measurement varies between 2 % and 6 % in the electron channel and between 1 % and 3 % in the muon channel for $N_{\mathrm {jets}}$ ranging from 1 to 4.

## Combination of electron and muon channels

In order to increase the precision of the $W\,\mathtt + \,\mathrm {jets}$ to $Z \,\mathtt + \,\mathrm {jets}$ differential cross-section ratio measurements the results obtained for each observable in the electron and the muon channels were statistically combined, accounting for correlations between the sources of systematic uncertainties affecting each channel. Since the electron and muon measurements were performed in different fiducial regions, bin-by-bin correction factors, estimated using ALPGEN Monte Carlo samples, were applied to each measured distribution to extrapolate the measurements to the common phase space defined in Table 1. The corrections to the $R_{\mathrm {jets}}$ measurement are of the order of $6\, \%$ in the electron channel and $1\, \%$ in the muon channel. The uncertainties on the acceptance corrections are below $0.5\, \%$, as determined by using SHERPA instead of ALPGEN. By comparing distributions computed at LO and NLO, it was checked with MCFM that NLO effects on the extrapolation to the common phase space are negligible. Before the combination was performed, the individual results of the two channels were compared to each other after extrapolation; the results are compatible within their respective uncertainties.

The method of combination used is an averaging procedure described in Refs. [45, 46]. The distributions for each observable were combined separately by minimising a $\chi ^2$ function which takes into account the results in the extrapolated electron and muon channels and all systematic uncertainties on both channels. The uncertainties on the modelling in the unfolding procedure, the integrated luminosity, the background contributions estimated from simulations except for $Z \,\mathtt + \,\mathrm {jets}$ and $W\,\mathtt + \,\mathrm {jets}$ backgrounds and systematic uncertainties on the data-driven $t\bar{t}$ estimation were treated as correlated among bins and between channels. The lepton systematic uncertainties were assumed to be correlated between bins of a given distribution, but uncorrelated between the two lepton channel measurements. The statistical uncertainties of the data, the statistical uncertainty of the unfolding procedure, and the statistical uncertainty of the $t\bar{t}$ fit were treated as uncorrelated among bins and channels. The systematic uncertainties on multi-jet backgrounds, which contain correlated and uncorrelated components, are also treated as uncorrelated among bins and channels. This choice has little impact on the final combined results and was chosen as it is slightly more conservative in terms of the total uncertainty of the combined results. Finally, the uncertainties from the jet energy scale, the jet energy resolution, the $E_{\mathrm {T}}^{\mathrm {miss}}$ calculation and the $Z \,\mathtt + \,\mathrm {jets}$ and $W\,\mathtt + \,\mathrm {jets}$ background contributions were treated as fully correlated between all bins and do not enter into the combination procedure to avoid numerical instabilities due to the statistical component in these uncertainties. For the combined results, each of these uncertainties was taken as the weighted average of the corresponding uncertainty on the electron and muon measurements, where the weights are the inverse of the sum in quadrature of all the uncorrelated uncertainties that entered in the combination.

## Theoretical predictions

The measured distributions of all the observables considered in the analysis are compared at particle level to various pQCD predictions in the phase space defined in Table 1.

Next-to-leading-order pQCD predictions were calculated with BlackHat+SHERPA  [47–49] at parton level for various parton multiplicities, from zero to four. In this calculation BlackHat is used for the computation of the virtual one-loop matrix elements, while SHERPA is used for the real emission part and the phase-space integration. The fixed-order calculation is performed at parton level only, without radiation and hadronization effects. Renormalisation and factorisation scales were evaluated at $H_\mathrm{T}/2$, where $H_\mathrm{T}$ is defined as the scalar sum of the transverse momenta of all stable particles in each event. The PDF set used was CT10 [17]. Partons were clustered into jets using the anti-$k_{t}$ algorithm with $R=0.4$.

The effect of uncertainties on the prediction has been computed for $R_{\mathrm {jets}}$, accounting for correlation between the individual $W\,\mathtt + \,\mathrm {jets}$ and $Z \,\mathtt + \,\mathrm {jets}$ processes. The uncertainties on the theoretical predictions are significantly reduced in this procedure, with the statistical uncertainty on the samples often dominating.

Uncertainties on the renormalisation and the factorisation scales were evaluated by varying these scales independently to half and twice their nominal value. The PDF uncertainties were computed from the CT10 eigenvectors, derived with the Hessian method at $68\,\%$ confidence level [17]. The changes in $R_{\mathrm {jets}}$ due to these PDF variations were combined and used as the uncertainty. In addition, the nominal value of the strong coupling constant, $\alpha _{s} = 0.118$, was varied by $\pm 0.0012$, and the impact of this variation was taken into account in the PDF uncertainty. All the uncertainty components mentioned above were then added in quadrature. The total systematic uncertainty on the prediction ranges from 0.3 % to 1.8 % for inclusive jet multiplicities ranging from one to four, and from 2 % to 6 % for leading-jet ${p}_\mathrm{T}$ ranging from $30\, {\mathrm {\ GeV}}$ to $700\,{\mathrm {\ GeV}}$.

In order to compare the BlackHat+SHERPA parton-level predictions with the measurements at particle level, a set of non-perturbative corrections was applied to the predictions. Corrections for the underlying event (UE) were calculated using samples generated with ALPGEN+HERWIG+JIMMY. The ratio of samples where the UE has been switched on and off was evaluated in each bin of each distribution. Corrections for the hadronization of partons to jets were computed using similar samples by forming the ratio of distributions obtained using jets clustered from hadrons versus jets clustered from partons. In $R_{\mathrm {jets}}$, the hadronization and UE corrections have opposite signs and are quite small (typically 2 % to 3 % for the exclusive jet multiplicity), so the overall correction factor is close to unity. Additional ALPGEN+PYTHIA samples were used to estimate the uncertainties due to these non-perturbative corrections, which are typically well below $1\, \%$.

Finally, corrections for QED final-state radiation were calculated as the ratio of $R_{\mathrm {jets}}$ derived from “dressed” leptons to $R_{\mathrm {jets}}$ defined before any final-state photon radiation, using ALPGEN samples interfaced to PHOTOS. These corrections range between 1 % and 2 % for both the electron and the muon channel. Systematic uncertainties were derived by comparing with corrections obtained using SHERPA, which calculates final-state QED radiation using the YFS method [50]. The differences between the predictions are typically well below 1 %.

Tree-level multi-leg matrix element calculations matched to parton showering algorithms were obtained from the ALPGEN and SHERPA generators. These calculations use different PDF sets, matching procedures, parton shower evolution, and hadronization and multi-parton interaction modelling, as detailed in Sect. 3. Only statistical uncertainties were considered for these predictions, which are compared with the BlackHat+Sherpa calculations and the data in Sect. 10.

## Results and discussion

The theoretical predictions described in Sect. 9 are compared to the experimental data unfolded to particle level, as defined in Sect. 6. Individual ratios of the BlackHat+SHERPA, ALPGEN, and SHERPA predictions to unfolded data make it possible to disentangle the important features of each theoretical prediction. The $R_{\mathrm {jets}}$ results highlight the ability of these Monte Carlo programs to model the differences between $Z \,\mathtt + \,\mathrm {jets}$ and $W\,\mathtt + \,\mathrm {jets}$ processes.

**Fig. 1:**
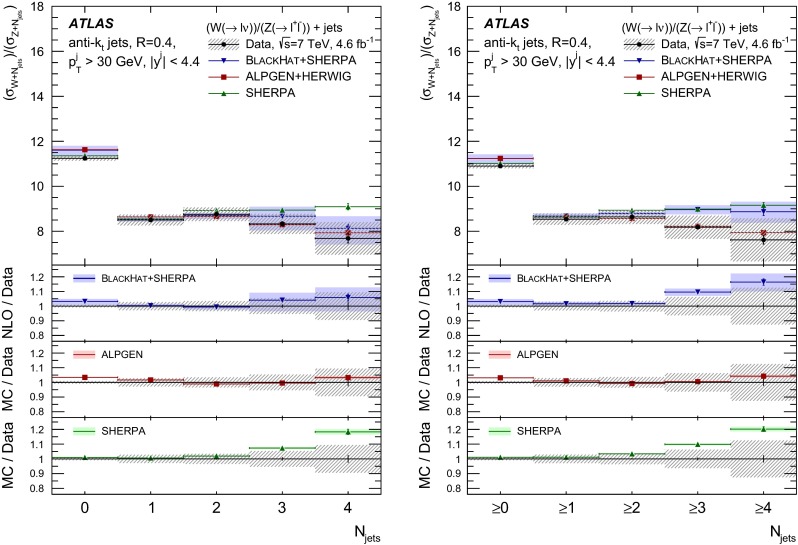
The ratio of $W\,\mathtt + \,\mathrm {jets}$ and $Z \,\mathtt + \,\mathrm {jets}$ production cross sections, $R_{\mathrm {jets}}$, as a function of exclusive jet multiplicity, ${N}_{\mathrm {jets}}$, (*left*) and inclusive jet multiplicity (*right*). The electron and muon channel measurements are combined as described in the text. Ratios of the BlackHat+SHERPA NLO calculation and the ALPGEN and SHERPA generators to the data are shown in the *lower panels*. *Vertical error bars* show the respective statistical uncertainties. The *hatched error band* shows statistical and systematic uncertainties added in quadrature for the data. The *solid error bands* show the statistical uncertainties for the ALPGEN and SHERPA predictions, and the combined statistical and theoretical uncertainties for the BlackHat+SHERPA prediction

Figure 1 shows $R_{\mathrm {jets}}$ as a function of exclusive and inclusive jet multiplicity. The values are detailed in Tables 6 and 7, respectively.^5^  The theoretical predictions describe the data fairly well, given the experimental uncertainties, with few exceptions. At high jet multiplicities, where the effects of hard QCD radiation are tested, the SHERPA prediction is about $1.5$ standard deviations ($1.5 \sigma$) of the experimental error greater than the measurement. BlackHat+SHERPA is able to describe $R_{\mathrm {jets}}$ measured as a function of exclusive jet multiplicity, within the theoretical uncertainties, although it is about $1\sigma$ greater than the measurement at high inclusive jet multiplicities; this is expected since it does not include all contributions for events with at least four jets.

**Table 6:** The ratio of $W\,\mathtt + \,\mathrm {jets}$ and $Z \,\mathtt + \,\mathrm {jets}$ production cross sections, $R_{\mathrm {jets}}$, as a function of exclusive jet multiplicity in the phase space defined in Table 1

$N_\mathrm {jets}$	$R_{\mathrm {jets}}$
= 0	11.24 $\pm$ 0.01 (stat.) $\pm$ 0.11 (syst.)
= 1	8.50 $\pm$ 0.02 (stat.) $\pm$ 0.24 (syst.)
= 2	8.76 $\pm$ 0.05 (stat.) $\pm$ 0.30 (syst.)
= 3	8.33 $\pm$ 0.10 (stat.) $\pm$ 0.44 (syst.)
= 4	7.69 $\pm$ 0.21 (stat.) $\pm$ 0.70 (syst.)

**Table 7:** The ratio of $W\,\mathtt + \,\mathrm {jets}$ and $Z \,\mathtt + \,\mathrm {jets}$ production cross sections, $R_{\mathrm {jets}}$, as a function of inclusive jet multiplicity in the phase space defined in Table 1

$N_\mathrm {jets}$	$R_{\mathrm {jets}}$
$\ge {0}$	10.90 $\pm$ 0.01 (stat.) $\pm$ 0.10 (syst.)
$\ge {1}$	8.54 $\pm$ 0.02 (stat.) $\pm$ 0.25 (syst.)
$\ge {2}$	8.64 $\pm$ 0.04 (stat.) $\pm$ 0.32 (syst.)
$\ge {3}$	8.18 $\pm$ 0.08 (stat.) $\pm$ 0.51 (syst.)
$\ge {4}$	7.62 $\pm$ 0.19 (stat.) $\pm$ 0.94 (syst.)

**Fig. 2:**
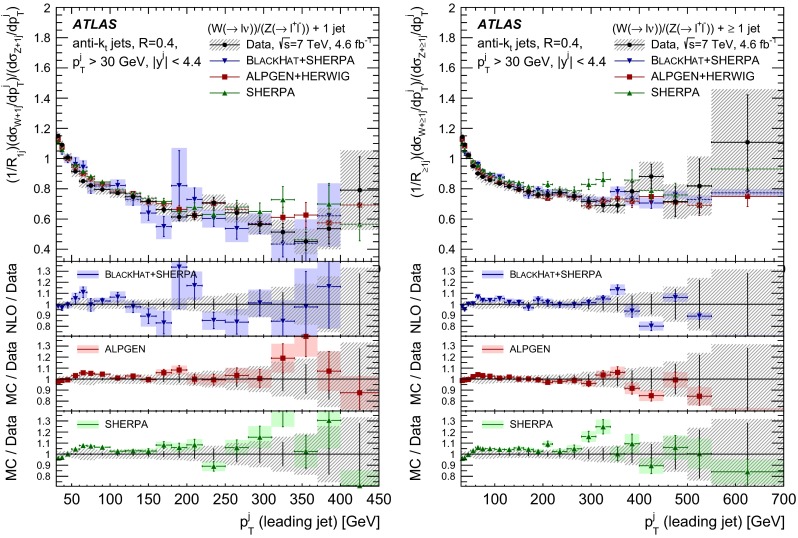
The ratio of $W\,\mathtt + \,\mathrm {jets}$ and $Z \,\mathtt + \,\mathrm {jets}$ production cross sections, $R_{\mathrm {jets}}$, normalized as described in the text versus the leading-jet transverse momentum, $p^\mathrm{j}_\mathrm{T}$, for ${N}_{\mathrm {jets}}$ $= 1$ ($left$) and ${N}_{\mathrm {jets}}$ $\ge 1$ (*right*). The electron and muon channel measurements are combined as described in the text. Ratios of the BlackHat+SHERPA NLO calculation and the ALPGEN and SHERPA generators to the data are shown in the *lower panels*. *Vertical error bars* show the respective statistical uncertainties. The *hatched error band* shows statistical and systematic uncertainties added in quadrature for the data. The *solid error bands* show the statistical uncertainties for the ALPGEN and SHERPA predictions, and the combined statistical and theoretical uncertainties for the BlackHat+SHERPA prediction

In the following figures, $R_{\mathrm {jets}}$ is normalized to the ratio of the $W$ and $Z$ cross sections in the corresponding jet multiplicity bin presented in Fig. 1, so that the shapes of the distributions can be compared. Figure 2 shows the $R_{\mathrm {jets}}$ ratio versus the leading-jet ${p}_\mathrm{T}$ for ${N}_{\mathrm {jets}} =1$ and ${N}_{\mathrm {jets}} \ge 1$. At low transverse momentum (${p}_\mathrm{T} <200\,{\mathrm {\ GeV}}$), the $R_{\mathrm {jets}}$ distribution falls as the leading-jet ${p}_\mathrm{T}$ increases, indicating that the shapes in $W\,\mathtt + \,\mathrm {jets}$ and $Z \,\mathtt + \,\mathrm {jets}$ events are different. This is due to the $W$ and $Z$ boson mass difference, which affects the scale of the parton radiation, and the different vector-boson polarizations, which affect the kinematics of their decay products. In the small region very close to the minimum value of the jet ${p}_\mathrm{T}$ considered in the analysis, where radiative parton shower effects play a major role, all of the predicted shapes exhibit trends different from those in the data, but the ALPGEN predictions still show the best agreement.

**Fig. 3:**
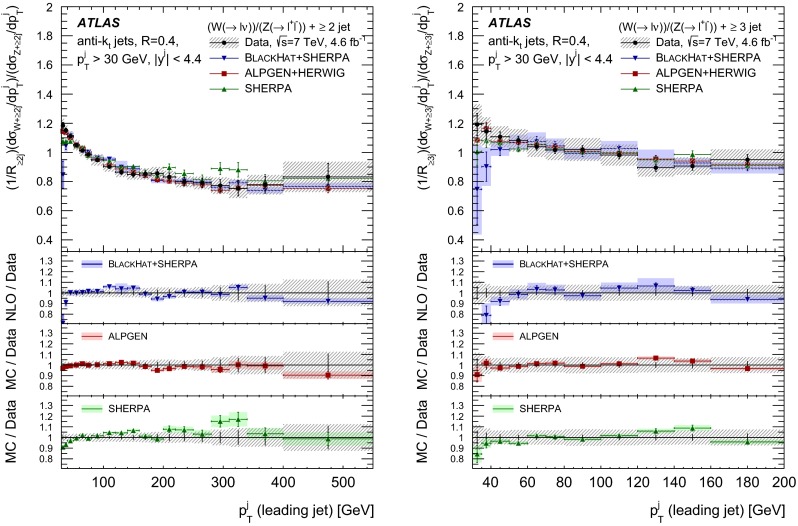
The ratio of $W\,\mathtt + \,\mathrm {jets}$ and $Z \,\mathtt + \,\mathrm {jets}$ production cross sections, $R_{\mathrm {jets}}$, normalized as described in the text versus the leading-jet transverse momentum, $p^\mathrm{j}_\mathrm{T}$, for ${N}_{\mathrm {jets}}$ $\ge 2$ (*left*) and $\ge 3$ (*right*). The electron and muon channel measurements are combined as described in the text. Ratios of the BlackHat+SHERPA NLO calculation and the ALPGEN and SHERPA generators to the data are shown in the *lower panels*. *Vertical error bars* show the respective statistical uncertainties. The *hatched error band* shows statistical and systematic uncertainties added in quadrature for the data. The *solid error bands* show the statistical uncertainties for the ALPGEN and SHERPA predictions, and the combined statistical and theoretical uncertainties for the BlackHat+SHERPA prediction

Figure 3 shows $R_{\mathrm {jets}}$ versus the leading-jet ${p}_\mathrm{T}$ for ${N}_{\mathrm {jets}}$ $\ge 2$ and ${N}_{\mathrm {jets}}$ $\ge 3$. The $R_{\mathrm {jets}}$ distribution falls less steeply the more jets are in the event. This is due to the smaller average vector-boson ${p}_\mathrm{T}$, which reduces the effects arising from differences in boson masses and polarizations. At the lowest ${p}_\mathrm{T}$ values considered the comparison with the data shows a tendency for different behaviour of the theoretical predictions, especially in events with at least three jets. The effect, which is most pronounced for BlackHat+SHERPA, is expected in case of lack of resummation of soft and collinear parton emissions, as in this calculation.

**Fig. 4:**
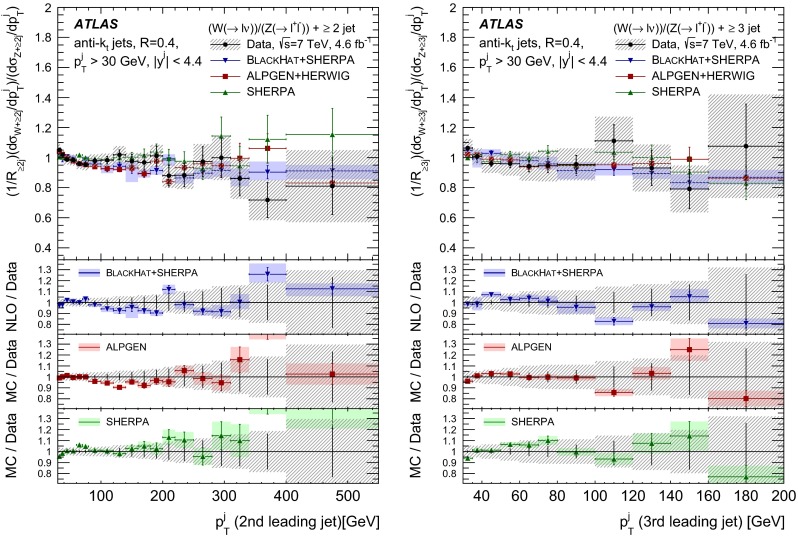
The ratio of $W\,\mathtt + \,\mathrm {jets}$ and $Z \,\mathtt + \,\mathrm {jets}$ production cross sections, $R_{\mathrm {jets}}$, normalized as described in the text versus the second-leading-jet transverse momentum, $p^\mathrm{j}_\mathrm{T}$, for ${N}_{\mathrm {jets}}$ $\ge$ 2 (*left*) and versus the third-leading-jet ${p}_\mathrm{T}$ for ${N}_{\mathrm {jets}}$ $\ge 3$ (*right*). The electron and muon channel measurements are combined as described in the text. Ratios of the BlackHat+SHERPA NLO calculation and the ALPGEN and SHERPA generators to the data are shown in the *lower panels*. *Vertical error bars* show the respective statistical uncertainties. The *hatched error band* shows statistical and systematic uncertainties added in quadrature for the data. The *solid error bands* show the statistical uncertainties for the ALPGEN and SHERPA predictions, and the combined statistical and theoretical uncertainties for the BlackHat+SHERPA prediction

Figure 4 shows $R_{\mathrm {jets}}$ versus the second- and third-leading-jet ${p}_\mathrm{T}$ for ${N}_{\mathrm {jets}}$ $\ge 2$ and ${N}_{\mathrm {jets}}$ $\ge 3$ respectively. The various predictions agree with the data distributions, given the uncertainties, except for small deviations in the second-leading-jet ${p}_\mathrm{T}$ for ${N}_{\mathrm {jets}} \ge 2$.

**Fig. 5:**
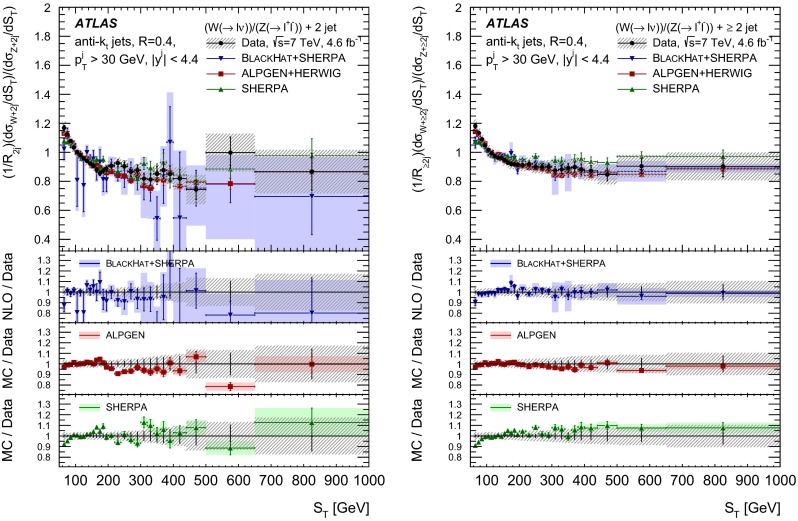
The ratio of $$W\,\mathtt + \,\mathrm {jets}$$ and $$Z \,\mathtt + \,\mathrm {jets}$$ production cross sections, $$R_{\mathrm {jets}}$$, normalized as described in the text versus the scalar sum $${p}_\mathrm{T}$$ of jets, $${S}_\mathrm{T}$$, for $${N}_{\mathrm {jets}}$$ $$ = 2$$ (*left*) and $$\ge $$ 2 (*right*). The electron and muon channel measurements are combined as described in the text. Ratios of the BlackHat+SHERPA NLO calculation and the ALPGEN and SHERPA generators to the data are shown in the *lower panels*. *Vertical error bars* show the respective statistical uncertainties. The *hatched error band* shows statistical and systematic uncertainties added in quadrature for the data. The *solid error bands* show the statistical uncertainties for the ALPGEN and SHERPA predictions, and the combined statistical and theoretical uncertainties for the BlackHat+SHERPA prediction

**Fig. 6:**
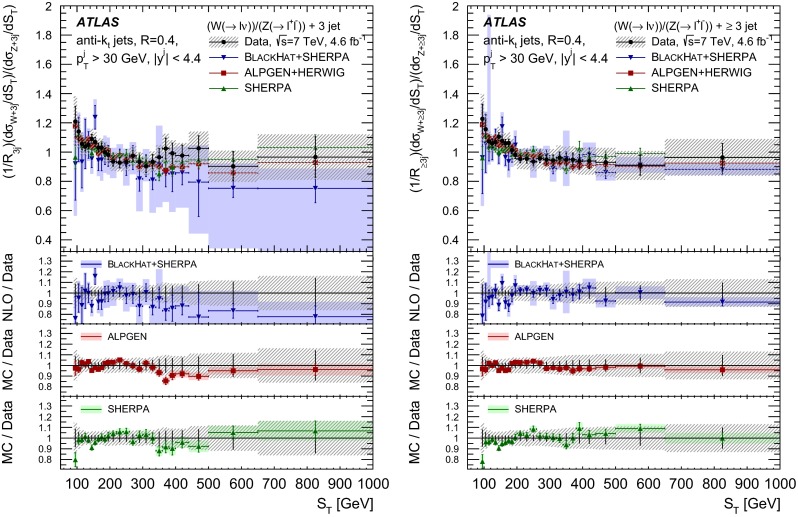
$$R_{\mathrm {jets}}$$ normalized as described in the text versus the scalar sum $${p}_\mathrm{T}$$ of jets, $${S}_\mathrm{T}$$ for $${N}_{\mathrm {jets}}$$ $$ = 3$$ (*left*) and $$\ge $$ 3 (*right*). The electron and muon channel measurements are combined as described in the text. Ratios of the BlackHat+SHERPA NLO calculation and the ALPGEN and SHERPA generators to the data are shown in the *lower panels*. *Vertical error bars* show the respective statistical uncertainties. The *hatched error band* shows statistical and systematic uncertainties added in quadrature for the data. The *solid error bands* show the statistical uncertainties for the ALPGEN and SHERPA predictions, and the combined statistical and theoretical uncertainties for the BlackHat+SHERPA prediction

The next kinematic observable studied is ${S}_\mathrm{T}$, the scalar sum of all jet transverse momenta in the event. This observable is often used in searches for new high-mass particles. Figure 5 shows $R_{\mathrm {jets}}$ versus ${S}_\mathrm{T}$ for ${N}_{\mathrm {jets}} = 2$ and ${N}_{\mathrm {jets}} \ge$ 2, while Fig. 6 shows $R_{\mathrm {jets}}$ versus ${S}_\mathrm{T}$ for ${N}_{\mathrm {jets}} = 3$ and ${N}_{\mathrm {jets}} \ge$ 3. At the lowest values of ${S}_\mathrm{T}$ the predicted distributions are different from the measured distributions, particularly for SHERPA, but in the higher-${S}_\mathrm{T}$ region the theoretical predictions describe the data well. The central value of the fixed-order BlackHat+SHERPA calculation does not reproduce the ${S}_\mathrm{T}$ distributions for $W\,\mathtt + \,\mathrm {jets}$ and $Z \,\mathtt + \,\mathrm {jets}$ separately as well as the inclusive calculation, corroborating the previous observations in Refs. [4, 5]. The tensions are due to the missing higher-order contributions which cancel almost completely in $R_{\mathrm {jets}}$.

**Fig. 7:**
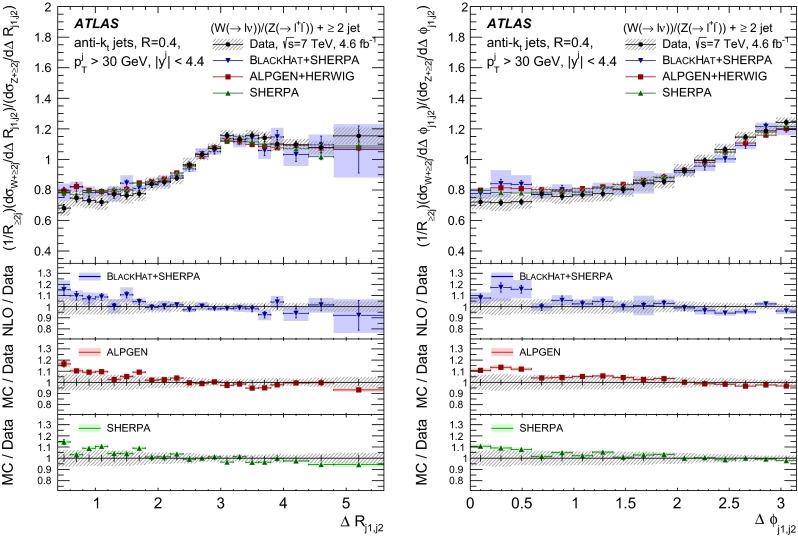
The ratio of $W\,\mathtt + \,\mathrm {jets}$ and $Z \,\mathtt + \,\mathrm {jets}$ production cross sections, $R_{\mathrm {jets}}$, normalized as described in the text versus the dijet angular separation, $\Delta R_\mathrm{j1,j2}$, (*left*) and the distance in $\phi$, $\Delta \phi _\mathrm{j1,j2}$, (*right*) for ${N}_{\mathrm {jets}}$ $\ge 2$. The electron and muon channel measurements are combined as described in the text. Ratios of the BlackHat+SHERPA NLO calculation and the ALPGEN and SHERPA generators to the data are shown in the *lower panels*. *Vertical error bars* show the respective statistical uncertainties. The *hatched error band* shows statistical and systematic uncertainties added in quadrature for the data. The *solid error bands* show the statistical uncertainties for the ALPGEN and SHERPA predictions, and the combined statistical and theoretical uncertainties for the BlackHat+SHERPA prediction

**Fig. 8:**
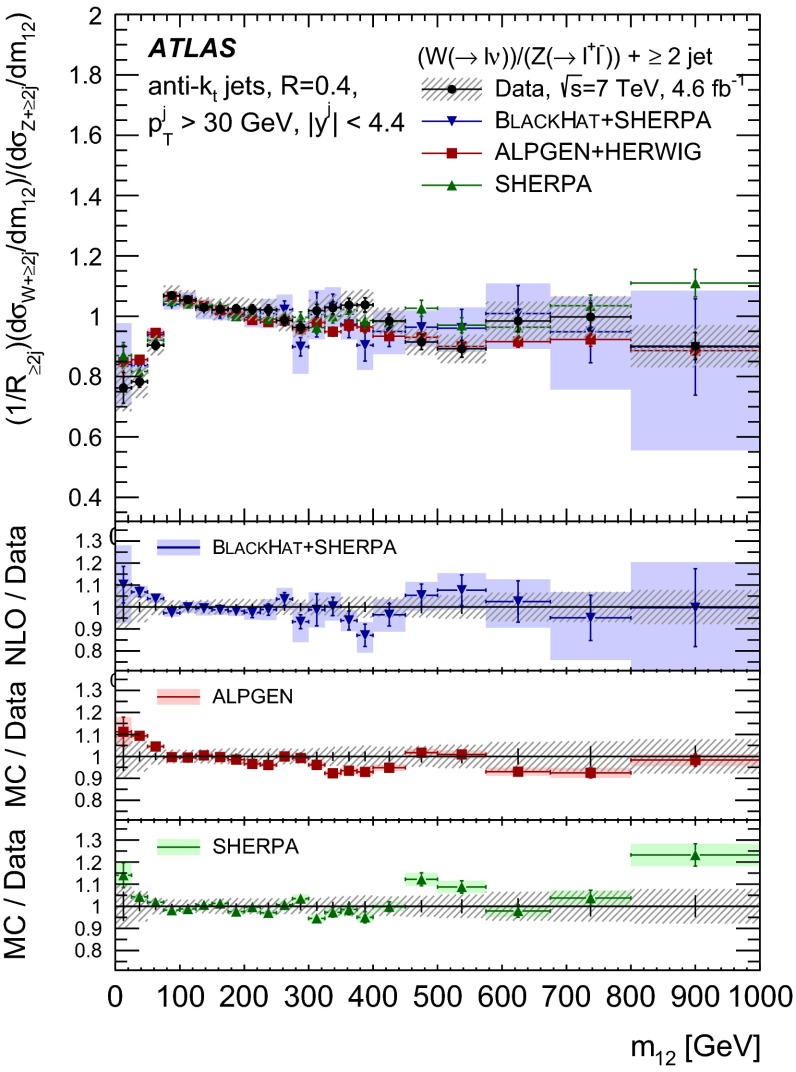
The ratio of $W\,\mathtt + \,\mathrm {jets}$ and $Z \,\mathtt + \,\mathrm {jets}$ production cross sections, $R_{\mathrm {jets}}$, normalized as described in the text versus the dijet invariant mass, $m_\mathrm{12}$, for ${N}_{\mathrm {jets}}$ $\ge 2$. The electron and muon channel measurements are combined as described in the text. Ratios of the BlackHat+SHERPA NLO calculation and the ALPGEN and SHERPA generators to the data are shown in the *lower panels*. *Vertical error bars* show the respective statistical uncertainties. The *hatched error band* shows statistical and systematic uncertainties added in quadrature for the data. The *solid error bands* show the statistical uncertainties for the ALPGEN and SHERPA predictions, and the combined statistical and theoretical uncertainties for the BlackHat+SHERPA prediction

Figure 7 shows the separation $\Delta R_\mathrm{j1,j2}$ and the azimuthal angular distance $\Delta \phi _\mathrm{j1,j2}$ between the two leading jets, and Fig. 8 shows their invariant mass $m_\mathrm{12}$ for ${N}_{\mathrm {jets}}$ $\ge$ 2. At the lowest $\Delta R_\mathrm{j1,j2}$ and $m_\mathrm{12}$ values, the predicted shapes differ from the measured ones. This is interpreted as a weak sensitivity to non-perturbative effects enhancing the difference in soft QCD radiation between $W$ and $Z$ events, but not cancelling completely in $R_{\mathrm {jets}}$.

**Fig. 9:**
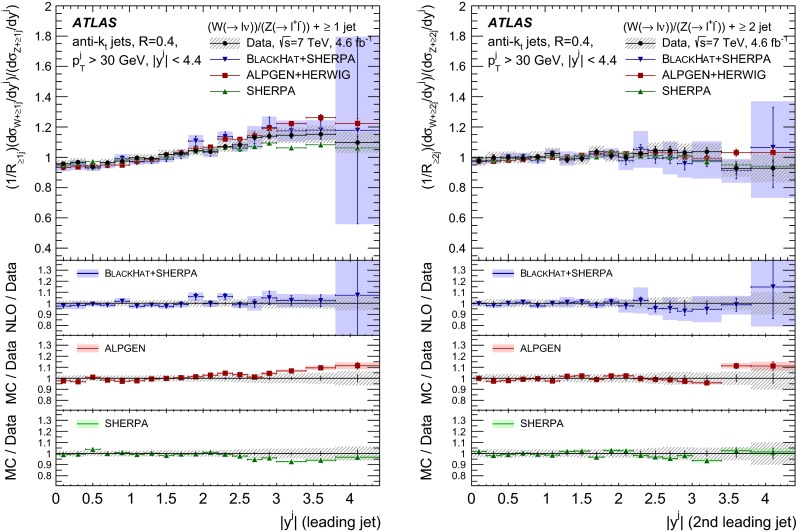
The ratio of $W\,\mathtt + \,\mathrm {jets}$ and $Z \,\mathtt + \,\mathrm {jets}$ production cross sections, $R_{\mathrm {jets}}$, normalized as described in the text versus the leading-jet rapidity, $y^j$, for ${N}_{\mathrm {jets}}$ $\ge 1$ (*left*) and second-leading-jet $y$ for ${N}_{\mathrm {jets}}$ $\ge 2$ (*right*). The electron and muon channel measurements are combined as described in the text. Ratios of the BlackHat+SHERPA NLO calculation and the ALPGEN and SHERPA generators to the data are shown in the *lower panels*. *Vertical error bars* show the respective statistical uncertainties. The *hatched error band* shows statistical and systematic uncertainties added in quadrature for the data. The *solid error bands* show the statistical uncertainties for the ALPGEN and SHERPA predictions, and the combined statistical and theoretical uncertainties for the BlackHat+SHERPA prediction

**Fig. 10:**
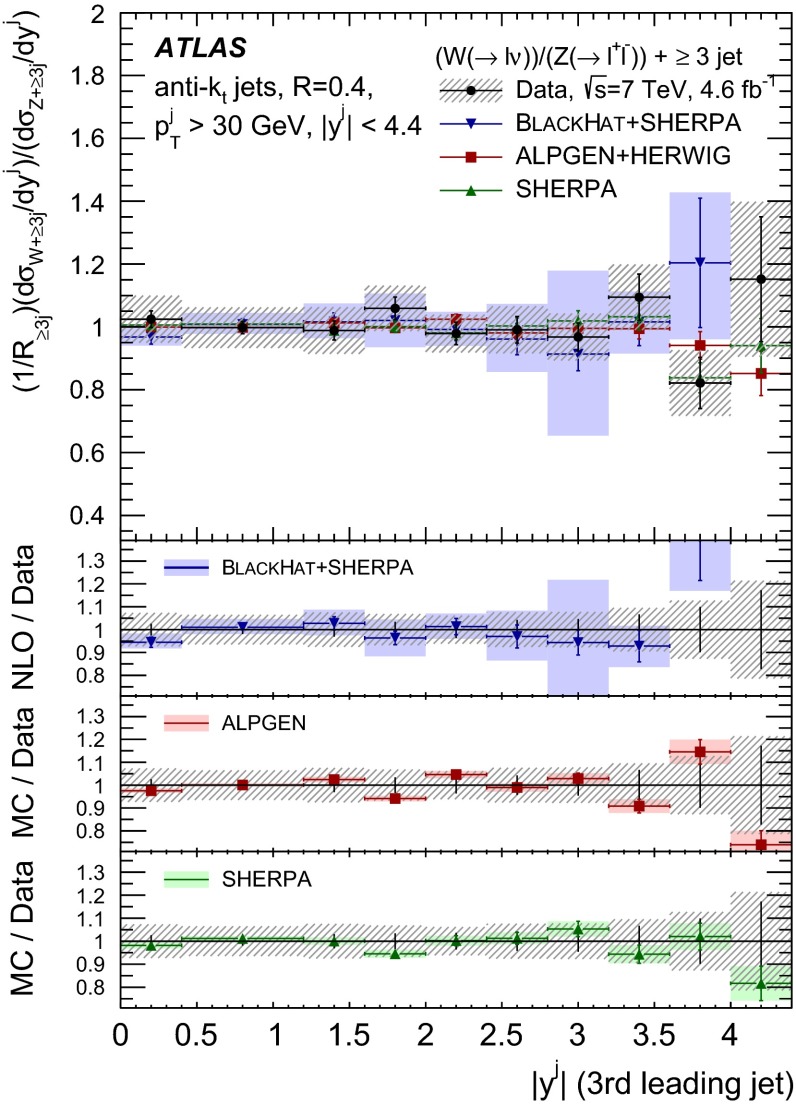
The ratio of $W\,\mathtt + \,\mathrm {jets}$ and $Z \,\mathtt + \,\mathrm {jets}$ production cross sections, $R_{\mathrm {jets}}$, normalized as described in the text versus the third-leading-jet rapidity, $y^j$, for ${N}_{\mathrm {jets}}$ $\ge$ 3. The electron and muon channel measurements are combined as described in the text. Ratios of the BlackHat+SHERPA NLO calculation and the ALPGEN and SHERPA generators to the data are shown in the *lower panels*. *Vertical error bars* show the respective statistical uncertainties. The *hatched error band* shows statistical and systematic uncertainties added in quadrature for the data. The *solid error bands* show the statistical uncertainties for the ALPGEN and SHERPA predictions, and the combined statistical and theoretical uncertainties for the BlackHat+SHERPA prediction

Figure 9 shows the leading-jet rapidity for ${N}_{\mathrm {jets}}$ $\ge 1$, and the second-leading-jet rapidity for ${N}_{\mathrm {jets}}$ $\ge 2$, while Fig. 10 shows the third-leading-jet rapidity for ${N}_{\mathrm {jets}}$ $\ge 3$. The different trends between predictions at high leading-jet rapidity can be due to the effects of the parton shower and, in some cases, different PDF sets. These effects, which do not cancel completely in $R_{\mathrm {jets}}$, are moderated by the presence of extra jets.

## Conclusions

Measurements of the ratio of $W\,\mathtt + \,\mathrm {jets}$ to $Z \,\mathtt + \,\mathrm {jets}$ production cross sections have been performed by the ATLAS experiment using a data sample of proton–proton collisions corresponding to an integrated luminosity of $4.6\,\mathrm {fb}^{-1}$ collected at a centre-of-mass energy of $\sqrt{s}=7\,{\mathrm {\ TeV}}$ at the LHC. The data were unfolded to particle level and compared to predictions from Monte Carlo simulations. By being sensitive to differences between $W\,\mathtt + \,\mathrm {jets}$ and $Z \,\mathtt + \,\mathrm {jets}$ events, and through large cancellations of experimental systematic uncertainties and non-perturbative QCD effects, the $R_{\mathrm {jets}}$ measurements provide information complementary to individual $W\,\mathtt + \,\mathrm {jets}$ and $Z \,\mathtt + \,\mathrm {jets}$ measurements. This $R_{\mathrm {jets}}$ measurement significantly improves on previous results by probing kinematic distributions for the first time in events with jet multiplicity up to four jets. It also allows a detailed comparison with state-of-the-art NLO pQCD Monte Carlo calculations, which agree well with the observed data except in a few specific regions. In particular, the BlackHat+SHERPA predictions for $R_{\mathrm {jets}}$ at high jet multiplicity and large leading-jet momenta are validated with this large dataset and are consistent with the results from tuned event generators. This new measurement highlights the success of recent theoretical advances and the opportunity for further tuning to improve the description of the production of vector bosons in association with jets.

## Footnotes

1 In this paper, $W$ means a $W^{+}$ or $W^{-}$ boson and $Z$ is defined as a $Z$ or $\gamma ^{*}$ boson.

2 ATLAS uses a right-handed coordinate system with its origin at the nominal interaction point (IP) in the centre of the detector and the $z$-axis along the beam pipe. The $x$-axis points from the IP to the centre of the LHC ring, and the $y$-axis points upward. Cylindrical coordinates ($r$, $\phi$) are used in the transverse plane, $\phi$ being the azimuthal angle around the beam pipe. The pseudorapidity is defined in terms of the polar angle $\theta$ as $\eta = -\ln \tan (\theta /2)$.

3 Angular separations between particles or reconstructed objects are measured in $\eta$–$\phi$ space using

$$\begin{aligned} \Delta R \equiv \sqrt{ (\Delta \phi )^{2} \mathtt + (\Delta \eta )^{2} }. \end{aligned}$$

4 The transverse mass of the $W$ boson is reconstructed as $m_T =\sqrt{2p_{\mathrm {T}}^{\ell }p_{\mathrm {T}}^{\nu }(1-\cos (\phi ^{\ell }-\phi ^{\nu }))}$ where $p_{\mathrm {T}}^{\ell }$ and $p_{\mathrm {T}}^{\nu }$ are the transverse momenta of the charged lepton and the neutrino respectively and $\phi ^{\ell }$ and $\phi ^{\nu }$ their azimuthal directions.

5 Tabulated values of the results are also available in the Durham HepData Project: http://hepdata.cedar.ac.uk.
